# Aqueous-Phase Photoreactions of Mixed Aromatic Carbonyl Photosensitizers Yield More Oxygenated, Oxidized, and less Light-Absorbing Secondary Organic Aerosol (SOA) than Single Systems

**DOI:** 10.1021/acs.est.3c10199

**Published:** 2024-04-23

**Authors:** Beatrix Rosette Go Mabato, Yong Jie Li, Dan Dan Huang, Chak K. Chan

**Affiliations:** † School of Energy and Environment, City University of Hong Kong, Tat Chee Avenue, Kowloon 999077, Hong Kong SAR, China; ‡ Department of Civil and Environmental Engineering, and Centre for Regional Ocean, Faculty of Science and Technology, University of Macau, Macau 999078, China; § Shanghai Academy of Environmental Sciences, Shanghai 200233, China; ∥ Division of Physical Sciences and Engineering, King Abdullah University of Science and Technology (KAUST), Thuwal, Jeddah 23955-6900, Kingdom of Saudi Arabia

**Keywords:** photosensitization, aromatic carbonyl photosensitizers, mixed photosensitizer system, aqueous secondary organic aerosol, light absorption, brown carbon (BrC)

## Abstract

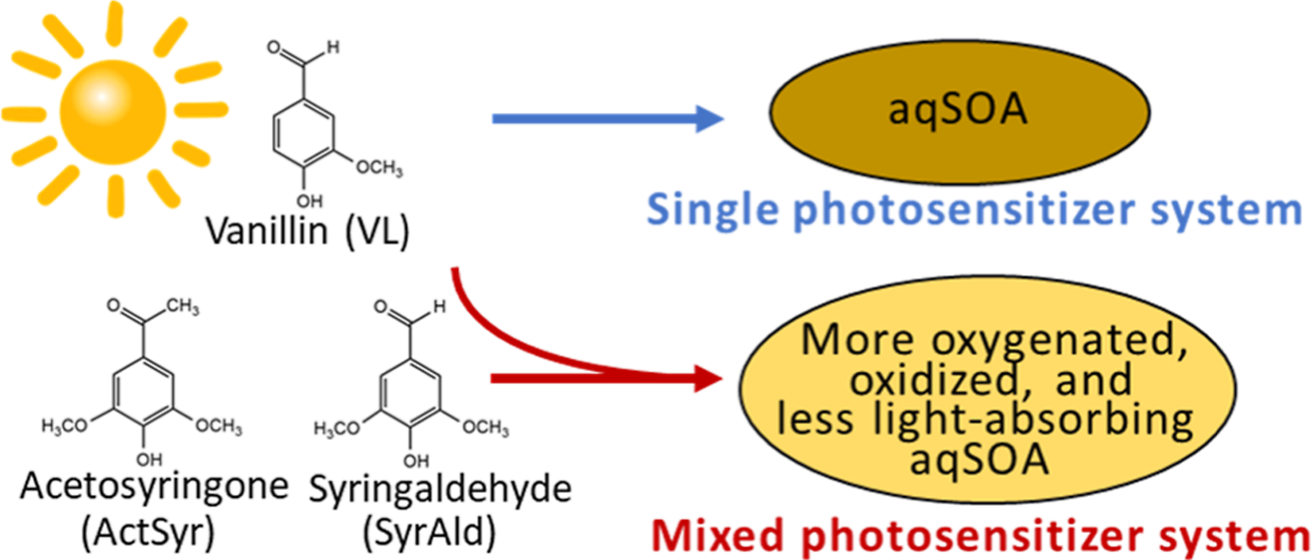

Aromatic carbonyls have been mainly probed as photosensitizers for aqueous secondary organic aerosol (aqSOA) and light-absorbing organic aerosol (i.e., brown carbon or BrC) formation, but due to their organic nature, they can also undergo oxidation to form aqSOA and BrC. However, photochemical transformations of aromatic carbonyl photosensitizers, particularly in multicomponent systems, are understudied. This study explored aqSOA formation from the irradiation of aromatic carbonyl photosensitizers in mixed and single systems under cloud/fog conditions. Mixed systems consisting of phenolic carbonyls only (VL + ActSyr + SyrAld: vanillin [VL] + acetosyringone [ActSyr] + syringaldehyde [SyrAld]) and another composed of both nonphenolic and phenolic carbonyls (DMB + ActSyr + SyrAld: 3,4-dimethoxybenzaldehyde [DMB], a nonphenolic carbonyl, + ActSyr + SyrAld) were compared to single systems of VL (VL*) and DMB (DMB*), respectively. In mixed systems, the shorter lifetimes of VL and DMB indicate their diminished capacity to trigger the oxidation of other organic compounds (e.g., guaiacol [GUA], a noncarbonyl phenol). In contrast to the slow decay and minimal photoenhancement for DMB*, the rapid photodegradation and significant photoenhancement for VL* indicate efficient direct photosensitized oxidation (i.e., self-photosensitization). Relative to single systems, the increased oxidant availability promoted functionalization in VL + ActSyr + SyrAld and accelerated the conversion of early generation aqSOA in DMB + ActSyr + SyrAld. Moreover, the increased availability of oxidizable substrates countered by stronger oxidative capacity limited the contribution of mixed systems to aqSOA light absorption. This suggests a weaker radiative effect of BrC from mixed photosensitizer systems than BrC from single photosensitizer systems. Furthermore, more oxygenated and oxidized aqSOA was observed with increasing complexity of the reaction systems (e.g., VL* < VL + ActSyr + SyrAld < VL + ActSyr + SyrAld + GUA). This work offers new insights into aqSOA formation by emphasizing the dual role of organic photosensitizers as oxidant sources and oxidizable substrates.

## Abstract 2

Aromatic carbonyl photosensitizers can contribute to aqueous secondary organic aerosol (aqSOA) formation as oxidant sources and oxidizable substrates. Compared to single systems, photoreactions of mixed aromatic carbonyl photosensitizers produce more oxygenated, oxidized, and less light-absorbing aqSOA.

## Introduction

Aqueous-phase photoreactions of aromatic carbonyl photosensitizers and phenols contribute to the aqueous secondary organic aerosol (aqSOA) and brown carbon (BrC) budget.^1−9^ BrC are organic aerosols that strongly absorb near-ultraviolet (UV) and visible light.^20^ Aromatic carbonyls and phenols are emitted by biomass burning (BB),^21−30^ photolysis of polycyclic aromatic hydrocarbons (PAHs),^31−33^ photochemical oxidation of aromatic compounds,^34−36^ automotive emissions,^37^ and laboratory-generated incense smoke.^38^ Several studies have explored the reactions of phenolic compounds mediated by various reactive species, including hydroxyl radicals (^•^OH),^39−42^ singlet oxygen (^1^O_2_),^43^ ozone (O_3_),^44,45^ reactive nitrogen species (e.g., ^•^NO and ^•^NO_2_),^15,46−49^ and triplet excited state of organic compounds (^3^C*).^1−13,15−18,50^ In particular, there has been a growing recognition of the importance of photosensitized phenol oxidation by ^3^C*, such as those from aromatic carbonyls.^1−13,15−18,50^ For example, relative to ^•^OH oxidation, ^3^C*-driven phenol oxidation has been reported to produce higher aqSOA mass yields at faster rates due to greater oxidant concentration in ^3^C*-initiated reaction.^2,5^ Moreover, we previously found that ring-opening products from ^3^C*-mediated phenol oxidation can react with ammonia to form N-containing products, including imidazoles,^15,16^ similar to the reactions between dicarbonyls (e.g., glyoxal) and ammonium/aminium salts.^51−58^

Vanillin (VL), 3,4-dimethoxybenzaldehyde (DMB), acetosyringone (ActSyr), and syringaldehyde (SyrAld) are aromatic carbonyl photosensitizers commonly emitted by BB and have been used as model compounds in aqSOA and BrC formation studies.^2,4−6,10,15,16,59^ Schauer et al. (2001) reported VL to be emitted at markedly higher rates from softwood (gymnosperms) combustion than from hardwood (angiosperms) combustion.^60^ By contrast, ActSyr and SyrAld were observed in hardwood combustion emissions but were not found at significant levels in softwood combustion emissions. VL and SyrAld are ubiquitous in fog and particulate matter present in BB-influenced urban and rural areas.^61−70^ Moreover, micromolar levels of VL and SyrAld have been detected in atmospheric waters.^23,26,71^ Furthermore, Rogge et al. (1998) documented emission rates of 22.62 mg kg^–1^ (pine wood) and 4.60 mg kg^–1^ (oak wood) for DMB.^27^

Photosensitized aqSOA formation has been primarily examined using reaction systems composed of an aromatic carbonyl photosensitizer (e.g., VL and DMB; a phenolic and a nonphenolic carbonyl, respectively) and a noncarbonyl phenol (e.g., guaiacol [GUA], syringol, and phenol) as the dominant aqSOA precursor.^2,4−6,8,9,12,15,16^ Aromatic carbonyl photosensitizers themselves can also be oxidized due to their organic nature.^4,15,30^ For instance, VL, DMB, ActSyr, and SyrAld have been reported to undergo transformation during the photo-oxidation of noncarbonyl phenols, with the resulting products likely containing low-volatility compounds.^4,6,15,16^ In addition, the direct photosensitized oxidation (i.e., oxidation of phenolic carbonyls by their ^3^C* or ^3^C*-derived secondary oxidants such as ^•^OH and ^1^O_2_, or self-photosensitization)^9,72^ of strongly light-absorbing phenolic carbonyls such as VL in single systems can be an effective aqSOA source.^4,15^ Nevertheless, while aqSOA formation via noncarbonyl phenol transformation has been studied exhaustively, discussion on the photochemical reactions of aromatic carbonyl photosensitizers remains minimal. Moreover, single-model photosensitizers are usually used in laboratory studies. However, considering the complexity of aerosol compositions, the existence of systems composed of mixed aromatic carbonyl photosensitizers is possible, but these have rarely been examined.^18^

**Figure 1:**
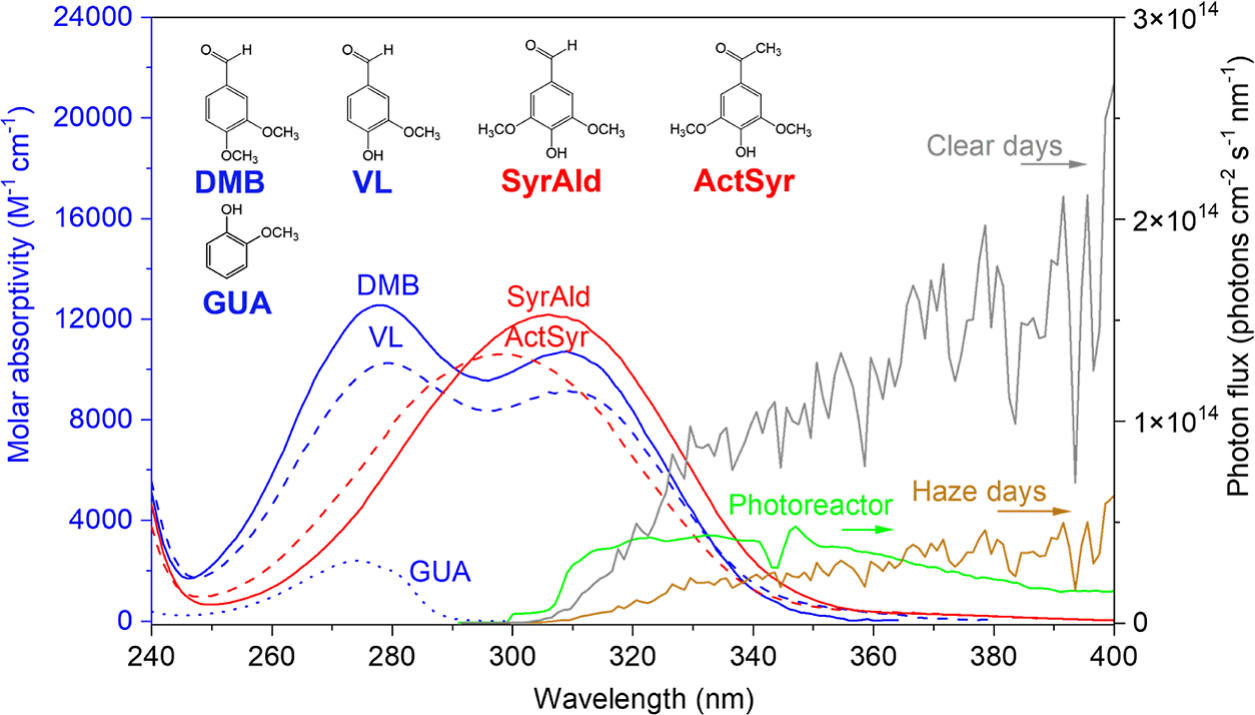
Base-10 molar absorptivities (M^–1^ cm^–1^) of DMB (blue solid line), VL (blue dashed line), SyrAld (red solid line), ActSyr (red dashed line), and GUA (blue dotted line). The green line is the photon flux in the aqueous photoreactor. The gray and brown lines are the photon fluxes on typical clear and haze days, respectively, in Beijing, China.^15,16^ The top of the figure also shows the structures of DMB, VL, SyrAld, ActSyr, and GUA.

The focus of this work is to simulate aqSOA formation from the photochemical reactions of mixed aromatic carbonyl photosensitizer systems in cloud/fogwater. As phenolic and nonphenolic carbonyls have similar concentrations in BB smoke,^24,26^ two model systems involving both classifications were considered: One was composed solely of phenolic carbonyls (VL, ActSyr, and SyrAld; hereafter VL + ActSyr + SyrAld) and another was composed of both nonphenolic and phenolic carbonyls (DMB, ActSyr, and SyrAld; hereafter DMB + ActSyr + SyrAld). VL and DMB (Henry’s law constants of 4.7 × 10^5^ and 7.3 × 10^3^ M atm^–1^, respectively)^30,73,74^ were chosen as the unique compounds in each system as their structures differ only by one functional group (−OH for VL and – OCH_3_ for DMB), and information on their photophysical properties (e.g., quantum yield of ^3^C* formation and ^3^C* lifetime) are available.^30^ ActSyr and SyrAld (Henry’s law constants of 1.1 × 10^6^ and 4.4 × 10^5^ M atm^–1^, respectively)^30,73,74^ are an aldehyde/ketone pair and differ only by a −CH_3_ group. This work also compared the photodegradation of VL and DMB (separately) in single photosensitizer systems termed VL* and DMB*, respectively, to that in VL + ActSyr + SyrAld and DMB + ActSyr + SyrAld. In addition, this study examined the behavior of mixed photosensitizer systems in the presence of GUA (denoted as VL + ActSyr + SyrAld + GUA and DMB + ActSyr + SyrAld + GUA, respectively), a noncarbonyl phenol significantly emitted by BB and found in both softwood and hardwood.^28,60,75^ Unlike the aromatic carbonyls studied here, GUA is not an effective photosensitizer because of its poor solar light absorption.^2,5^ The comparison of VL and DMB as photosensitizers for aqSOA formation via GUA oxidation has been studied in detail in our previous work.^16^Figure 1 shows the structures and molar absorptivities of the compounds studied, the photon flux in the aqueous photoreactor, and the photon fluxes on typical clear and haze days in Beijing, China. The decay rate constants, effective quantum yields, and atmospheric lifetimes were first determined, followed by measurement of absorbance changes and then analysis of the detected products and product distribution.

## Materials and Methods

### Aqueous-Phase Photochemical Experiments

The photochemical experiments were carried out using a photoreactor with a quartz window and a xenon lamp (model 6258; ozone-free xenon lamp; 300W; Newport) equipped with a long-pass filter (20CGA 305 nm cut-on filter; Newport) to eliminate light below 300 nm, similar to our previous works.^15,16^ The averaged initial photon flux in the reactor from 300 to 380 nm was ∼3 × 10^15^ photons cm^–2^ s^–1^ nm^–1^, comparable to our earlier studies.^15,16^ Solutions were prepared using 0.01 mM each of ActSyr (TCI, >98%), SyrAld (Sigma-Aldrich, 98%), and VL (Acros Organics, 99%, pure) or DMB (Acros Organics, 99+%), in the absence and presence of 0.01 mM GUA (Sigma-Aldrich, ≥98.0%). Solutions composed of 0.01 mM VL or DMB only were also prepared to represent single photosensitizer systems. These concentrations of aromatic carbonyls and GUA align with their expected levels in cloud/fogwater.^26,27,76^ Compared to the typical concentration of phenols used in earlier studies (0.1 mM),^5,6,15,16^ the lower concentration used here (0.01 mM each) is more relevant for concentrations of individual phenols estimated in cloudwater.^26,49^ The pH of the solutions was adjusted to 4, which falls within typical cloud pH values (2–7),^77^ using sulfuric acid (H_2_SO_4_; Acros Organics, ACS reagent, 95% solution in water). In addition, as the ionic strength for all conditions is low and within the same order of magnitude (10^–4^ mol kg^–1^), it is not anticipated to affect the reactions significantly. Table S1 lists the experiments performed in this work. Note that the identification and quantification of specific oxidants in the reaction systems examined are outside the scope of this study. The solutions were bubbled with synthetic air (0.5 dm^3^ min^–1^) for 30 min before irradiation and throughout the experiments to ensure air-saturated conditions^9,78^ and were consistently magnetically stirred. The reactions studied can generate ^3^C* and secondary oxidants (^1^O_2_, ^•^OH, and superoxide or hydroperoxyl radicals, O_2_^•–^/^•^HO_2_),^9,72^ but not ozone.

### Offline Chemical and Optical Analyses

The experiments were repeated independently at least three times. Samples were taken every 30 min for 180 min for offline analyses of (1) aromatic carbonyls and GUA concentrations using ultrahigh-performance liquid chromatography with a photodiode array detector (UHPLC-PDA) and (2) absorbance changes using UV–vis spectrophotometry. Samples taken before and after irradiation (180 min) were examined for (3) reaction products using UHPLC coupled with heated electrospray ionization Orbitrap mass spectrometry (UHPLC-HESI-Orbitrap-MS) operated in positive and negative ion modes and (4) concentrations of small organic acids using ion chromatography (IC; for VL + ActSyr + SyrAld and DMB + ActSyr + SyrAld only). A duration of 180 min was selected for irradiation, as it proved adequate to demonstrate variations in the reaction extent of the photosensitizers. Consequently, the reported product distributions pertain to products generated after a fixed time of photosensitization. It is stressed that the reported signal-weighted distributions were not an absolute comparison of the contributions of different product classifications to the total signals but rather a relative comparison. These were calculated by normalizing the peak area of each product to the total signal areas. The comparison of relative product abundance based on peak areas from mass spectrometry data has been used in numerous past studies to elucidate the relative importance of different types of compounds.^79−86^ However, as ionization efficiencies in soft ionization (e.g., ESI) may vary substantially among various compounds, uncertainties may be present when comparing the peak areas among different compounds.^87−91^ In this study, the detected compounds were assumed to have equal ionization efficiencies, which is typical for estimating the O/C ratios of SOA.^92−94^ Also, it should be noted that the UHPLC-PDA chromatograms of the detected products did not exhibit distinguishable peaks, which could be due to the chromophores having concentrations lower than the PDA detection limit. The pseudo-first-order decay rate constants (*k*′) were determined by fitting the exponential decay of the compounds studied. Note that data fitting was conducted in the initial linear region. The estimated *k*′, absorbance changes, and small organic acids concentration were averaged from triplicate experiments, and the reported errors represent 1 standard deviation. For UHPLC-HESI-Orbitrap-MS, two independently prepared samples for each reaction condition were analyzed. Only the reproducibly observed peaks in both sets of samples were considered. As the irradiation of DMB alone (DMB*) did not result in a statistically significant loss and absorbance changes (*p* > 0.05), MS analysis was not performed for this reaction condition. The Supporting Information (Sections S1–S6) presents details on the analytical procedures and calculation of effective quantum yields (Φ_e_) and estimated atmospheric lifetimes (τ). It should be emphasized that the reported *k*′ values were not corrected for the light screening effect to reflect the real decay rate constants in our experiments. Nonetheless, the light screening effect or light competition among the aromatic carbonyl photosensitizers was already considered in the estimation of Φ_e_ and τ by including *f*_*i*_(λ) or the relative light absorption, which is the fraction of light absorption by an aromatic carbonyl photosensitizer at a specific wavelength, calculated from its absorptivity and concentration.

## Results and Discussion

### Kinetic Analysis

**Figure 2:**
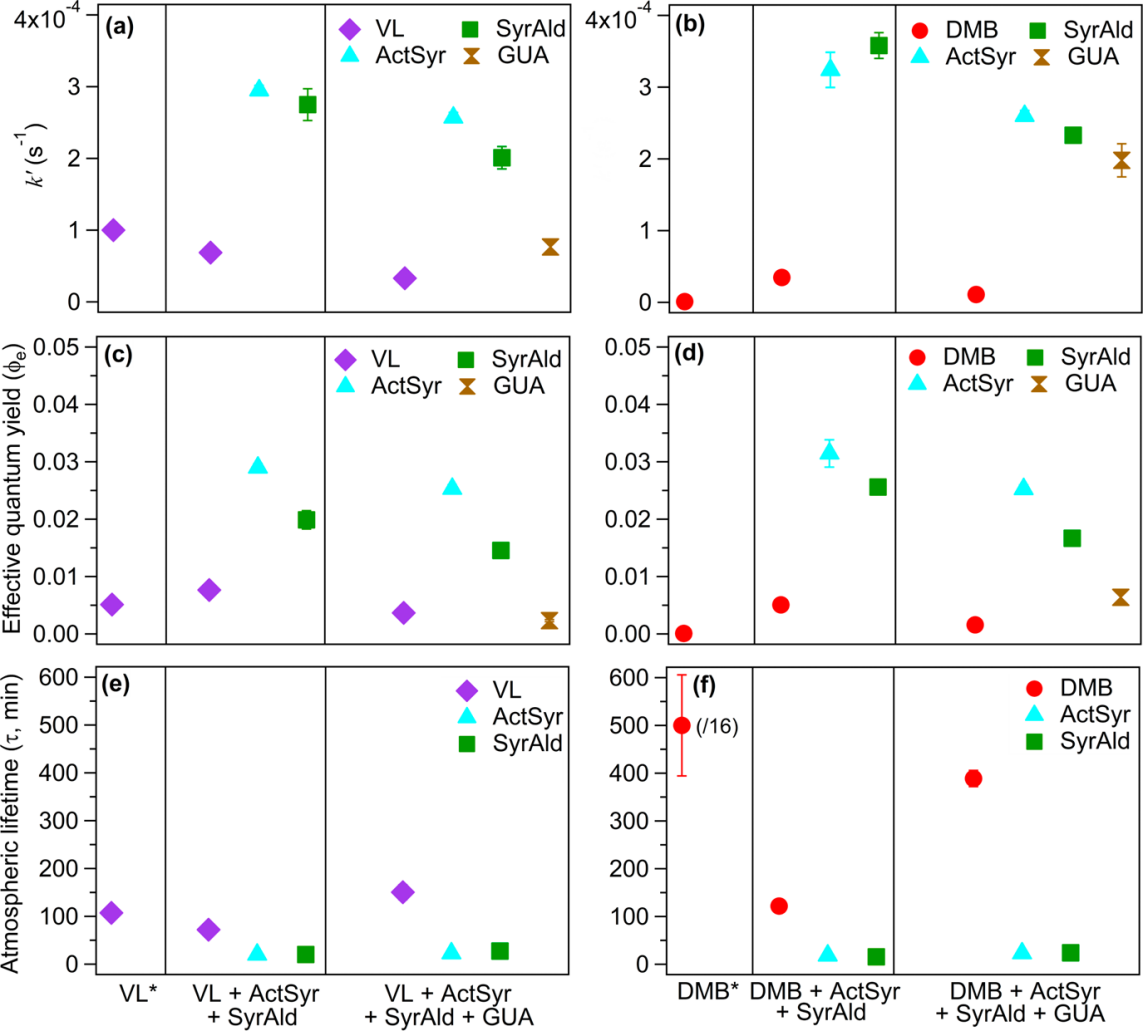
Initial decay rate constants (*k*′; a and b), effective quantum yields (Φ_e_; c and d), and estimated atmospheric lifetimes (τ; e and f) for VL, DMB, ActSyr, SyrAld, and GUA (a, c, and e: VL*, VL + ActSyr + SyrAld, and VL + ActSyr + SyrAld + GUA; b, d, and f: DMB*, DMB + ActSyr + SyrAld, and DMB + ActSyr + SyrAld + GUA). Note the scaling used for DMB* in panel f. Error bars represent 1 standard deviation of triplicate experiments; most error bars are smaller than the markers.

We first compared VL and DMB photodegradation in mixed systems (VL + ActSyr + SyrAld and DMB + ActSyr + SyrAld, respectively) to that in single systems (VL* and DMB*, respectively). Following that, we examined the behavior of mixed systems in the presence of GUA (VL + ActSyr + SyrAld + GUA and DMB + ActSyr + SyrAld + GUA). Figure 2 summarizes the parameters used for kinetic analysis: pseudo-first-order decay rate constants (*k*′), effective quantum yields (Φ_e_), and estimated atmospheric lifetimes (τ). Figure S1 exhibits representative kinetics plots from the irradiation of mixed photosensitizer systems. No significant decay of aromatic carbonyls was noted in the dark experiments (*p* > 0.05). Previous studies have reported the direct photosensitized oxidation of individual phenolic carbonyl photosensitizers (i.e., oxidation of phenolic carbonyls by their ^3^C* or secondary oxidants, or self-photosensitization)^4,15^ such as the case of VL* (Figure 2a). The direct photosensitized oxidation of individual phenolic carbonyl photosensitizers, which can proceed via H atom abstraction or electron transfer, is an efficient aqSOA formation pathway.^4,15^ The higher  in VL* (1.5 times; *p* < 0.05) than in VL + ActSyr + SyrAld can be attributed to the competition among the phenolic carbonyls in the latter for incoming photon fluxes, which would lessen ^3^VL* generation. As stated earlier, the *f*_*i*_(λ) or the relative light absorption (see eqs S6–S8) was used to quantify the light screening effect or light competition among the aromatic carbonyl photosensitizers in this work. For instance, the *f*_*i*_(λ) values for VL* and VL in VL + ActSyr + SyrAld are 1 and 0.27, respectively. Moreover, the *f*_*i*_(λ) was included in calculating Φ_e_ and τ to consider the light screening effect or light competition. The τ value of VL in VL* was 1.79 ± 0.08 h, comparable to a previous report.^18^ Nonetheless, in VL + ActSyr + SyrAld, the higher Φ_e_ and shorter τ of VL (both by 1.5 times; *p* < 0.05) than in VL* (Figure 2c,e) indicate that the reactions of ground-state VL with ^3^C* (and secondary oxidants) from ActSyr and SyrAld are more important than light competition. Establishing the contribution of specific oxidants to the reactions is, however, beyond the scope of this work.

Conversely, direct photosensitized oxidation does not appear important for the direct photodegradation of DMB (DMB*), a nonphenolic carbonyl. Instead, the recombination of ^3^DMB*-derived radicals outweighing further DMB transformation can explain the much lower  in DMB* (by 33 times) than in DMB + ActSyr + SyrAld (Figure 2b). The photoexcitation of aromatic ketones and benzaldehydes has been proposed to trigger their breakdown via Norrish reactions to form phenyl and benzoyl radicals, which can recombine to generate dimers and dimethoxybenzene.^11,95−99^ Such reactions can also occur for DMB. However, based on the low  and Φ_e_ (Figure 2b,d), as well as the minimal light absorption increase for DMB* (see Figure 5 and corresponding discussions), the radicals generated upon photoexcitation mainly underwent recombination to regenerate DMB, with further transformation reactions being less significant. On a related note, Yu et al. (2014) did not observe aqSOA formation from the irradiation of DMB alone.^5^ The τ value of DMB in DMB* was approximately 6 days, which is on the same order of magnitude as the value estimated from DMB irradiation on silica particles (about 2 days).^100^ In our previous work on photosensitized GUA oxidation by ^3^DMB* and ^3^VL* (separately), the faster consumption of VL than DMB was attributed to the −OH group of VL, rendering it more prone to oxidation and more reactive toward electrophilic aromatic substitution.^16^ Nonetheless, further work is needed to better understand the effect of different functional groups on the reactivity of aromatic carbonyl photosensitizers.

For DMB + ActSyr + SyrAld, the higher  was indicative of enhanced DMB transformation reactions in the presence of ActSyr and SyrAld. For instance, the phenyl and benzoyl radicals from DMB dissociation likely formed coupling products with intermediates derived from ActSyr and SyrAld. Moreover, DMB can also react with other oxidants such as ^•^OH,^11,13^ which can be formed upon ActSyr and SyrAld oxidation.^26^ In addition, ^3^DMB* and secondary oxidants possibly participated in subsequent reactions, as suggested by the more oxidized products from DMB + ActSyr + SyrAld than those from VL + ActSyr + SyrAld (see later discussions). The promoted DMB transformation reactions potentially account for the higher Φ_e_ and shorter τ value (both by ∼65 times) of DMB in DMB + ActSyr + SyrAld compared to DMB* (Figure 2d,f). SyrAld and ActSyr had comparable decay rate constants in VL + ActSyr + SyrAld (and DMB + ActSyr + SyrAld) (Figure 2a,b), which can be ascribed to the greater light absorption by SyrAld but the higher quantum yield for photodegradation for ActSyr, as reported by an earlier work.^4^ Moreover, among the phenolic carbonyls studied here, ActSyr and SyrAld had the highest *k*′, in line with additional −OCH_3_ groups in phenols, reducing their electron oxidation potential and thus enhancing their oxidation rate constants.^2,101^

The addition of GUA to VL + ActSyr + SyrAld and DMB + ActSyr + SyrAld resulted in 1.2 to 3.2 times lower *k*′ and Φ_e_ for the aromatic carbonyls (Figure 2a–d), with the greatest decrease observed for VL and DMB. This is ascribable to GUA and ground-state phenolic carbonyls competing for reactions with ^3^C* and increased rate of ^3^C* transition back to the ground state through GUA oxidation.^2,15,26^ We previously reported that  via oxidation by ^3^DMB* was ∼4 times higher than that by ^3^VL*.^16^ Similarly, the  and Φ_e_ for DMB + ActSyr + SyrAld + GUA were higher (by 2.3 and 2.7 times, respectively) than those for VL + ActSyr + SyrAld + GUA, attributable to DMB having a stronger photosensitizing ability^30^ and photostability than VL.^16^ DMB is considered to have a stronger photosensitizing ability than VL due to its higher quantum yield of ^3^C* formation and longer lifetime of ^3^DMB* than ^3^VL*.^30^ Moreover, the stronger photostability of DMB than VL is based on the minimal Φ_e_ of DMB in DMB*, which contrasts with the high Φ_e_ of VL in VL*, as discussed above, and our previous observations that VL decayed faster than DMB during GUA oxidation.^16^

In summary, VL photodegradation in VL* occurred rapidly, efficiently forming aqSOA. Conversely, DMB* demonstrated minimal DMB decay, indicating its low propensity for generating aqSOA. VL and DMB would yield enhanced aqSOA formation when present in mixed systems rather than in single systems. In addition, the shorter lifetimes of VL and DMB in mixed systems denote their lessened capacity to drive the oxidation of other organic compounds (e.g., noncarbonyl phenols). Moreover, aromatic carbonyl photosensitizers decayed more slowly and showed a lower transformation efficiency in the presence of GUA, reducing their contribution to aqSOA generation. ActSyr, SyrAld, and GUA decayed faster in DMB + ActSyr + SyrAld + GUA than in VL + ActSyr + SyrAld + GUA, ascribable to the stronger photosensitizing ability and photostability of DMB than VL.

### Product Distributions and Chemical Characteristics

**Figure 3:**
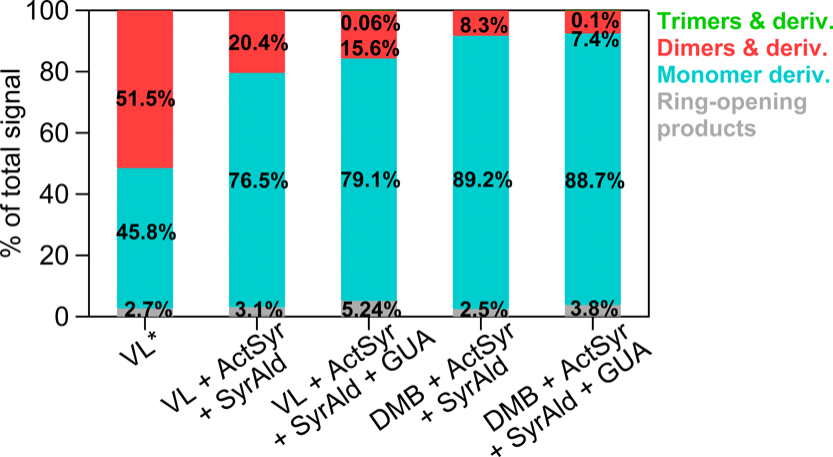
Signal-weighted distributions of VL*, VL + ActSyr + SyrAld, VL + ActSyr + SyrAld + GUA, DMB + ActSyr + SyrAld, and DMB + ActSyr + SyrAld + GUA aqSOA. These product distributions were calculated from combined UHPLC-HESI-Orbitrap-MS data obtained in positive and negative ion modes. The values indicate the contribution of different product classifications to the total signals for each reaction.

The aqSOA in this study are represented by the products detected using UHPLC-HESI-Orbitrap-MS. Note that as certain products may be volatile and would not be present in the particle phase upon evaporation, the detected species do not all necessarily contribute to aqSOA from the reactions examined. Nonetheless, an earlier study on photosensitized aqSOA formation from phenolic compounds reported that dissolved volatile and semivolatile species generated during the reactions accounted only for a small fraction (<10%) of the total carbon initially present in the reactants.^5^Figure 3 depicts the signal-weighted distributions of aqSOA calculated from combined positive and negative ion mode MS results. Figures S2 and S3 show the separate signal-weighted distributions for positive and negative ion mode MS results. The main aqSOA formation processes in both single and mixed photosensitizer systems were functionalization and oligomerization. For all mixed systems, monomer derivatives were the main contributors to product signals, followed by dimers, suggesting that functionalization prevailed over oligomerization within the experimental time scale. This contrasts with the majority of earlier investigations on aqSOA formation from binary systems of phenol with ^3^VL* or ^3^DMB* only,^5,8,10,15,16^ which have reported the predominance of oligomerization over functionalization. In those studies, the comparatively high initial phenol concentration (≥0.1 mM) used favored oligomerization. By contrast, the maximum concentration of phenolic compounds used in this study was 0.04 mM. As mentioned in the Materials and Methods Section, the lower concentration in this work is more relevant for concentrations of individual phenols estimated in cloudwater.^26,49^

**Figure 4:**
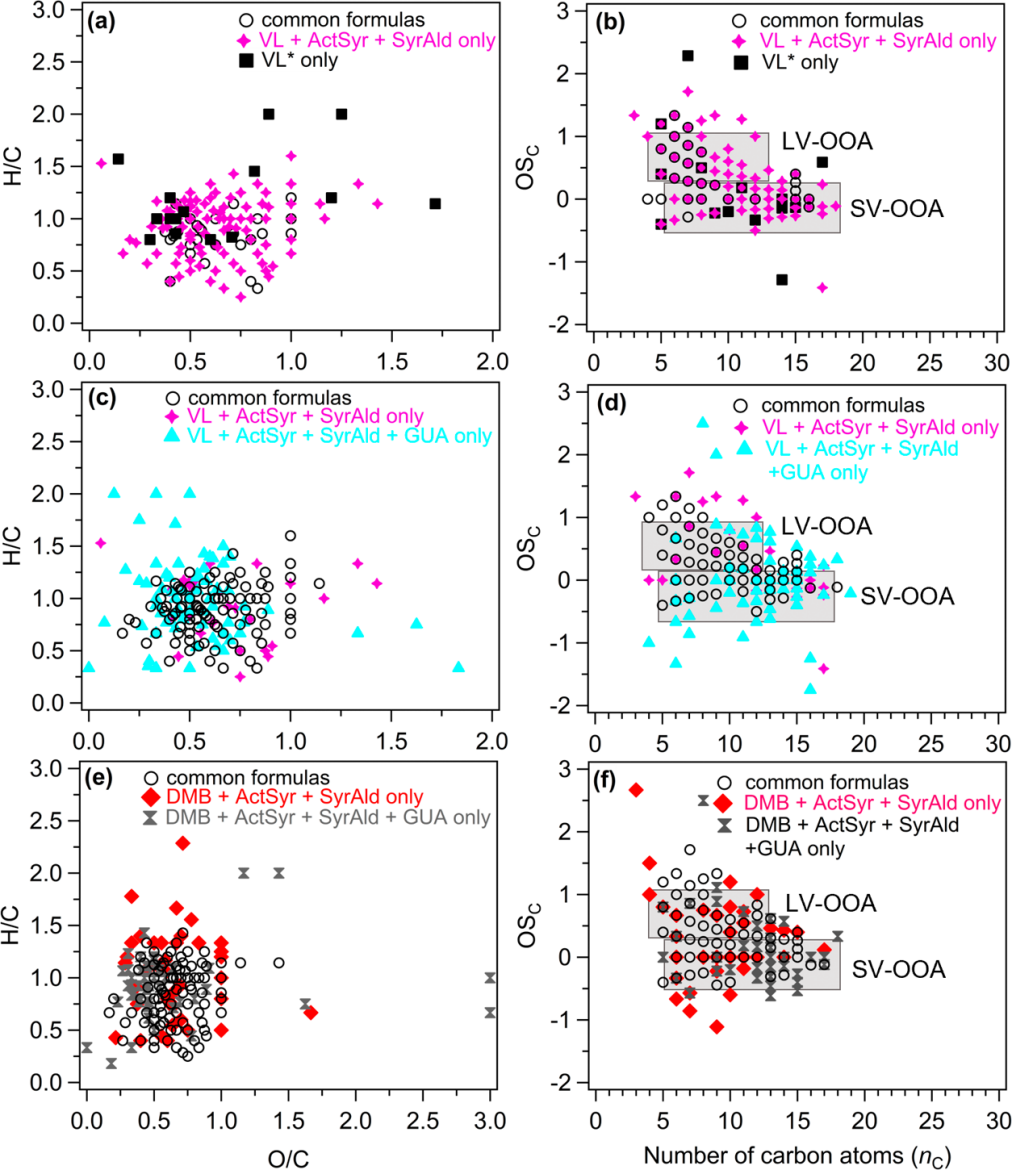
(a, c, and e) Van Krevelen diagrams and (b, d, and f) plots of the carbon oxidation state (OS_C_) vs the number of carbon atoms (*n*_C_) of common (empty black circles) and unique formulas detected from (a, b) VL + ActSyr + SyrAld (pink four corner stars) and VL* (black squares); (c, d) VL + ActSyr + SyrAld (pink four corner stars) and VL + ActSyr + SyrAld + GUA (cyan triangles); and (e, f) DMB + ActSyr + SyrAld (red diamonds) and DMB + ActSyr + SyrAld + GUA (gray hourglasses) based on combined UHPLC-HESI-Orbitrap-MS data obtained in positive and negative ion modes. The shaded areas (b, d, and f) indicate the regions corresponding to low-volatility oxygenated organic aerosol (LV-OOA) and semivolatile oxygenated organic aerosol (SV-OOA).^102^

Table 1 presents the chemical characteristics of the compounds studied and aqSOA detected. As the complexity of the system progresses (e.g., VL* < VL + ActSyr + SyrAld < VL + ActSyr + SyrAld + GUA), products exhibited a greater extent of oxygenation and oxidation, as suggested by higher ⟨O/C⟩ and average carbon oxidation state, ⟨OS_C_⟩. Figure 4 summarizes the van Krevelen diagrams and plots of the OS_C_ vs the number of carbon atoms (*n*_C_) of common and unique formulas between VL + ActSyr + SyrAld and VL*; VL + ActSyr + SyrAld, and VL + ActSyr + SyrAld + GUA; and DMB + ActSyr + SyrAld and DMB + ActSyr + SyrAld + GUA. Products from all reaction systems can be mainly classified as low-volatility oxygenated organic aerosol (LV-OOA) and semivolatile oxygenated organic aerosol (SV-OOA).^102^ The discussions for the major products (Tables S2–S6), van Krevelen diagrams showing the relative product abundance (Figure S4), and OS_C_ vs *n*_C_ plots showing the relative abundance and double bond equivalent (DBE) values (Figure S5) are available in the Supporting Information (Sections S7 and S8).

**Table 1:** Chemical Characteristics of the Compounds Studied and aqSOA From Each Experiment

compounds studied	O/C	H/C	OS_C_
(VL; C_8_H_8_O_3_)	0.38	1.00	–0.25
(DMB; C_9_H_10_O_3_)	0.33	1.11	–0.44
(ActSyr; C_10_H_12_O_4_)	0.40	1.20	–0.40
(SyrAld; C_9_H_10_O_4_)	0.44	1.11	–0.22
(GUA; C_7_H_8_O_2_)	0.29	1.14	–0.57

	reaction conditions	⟨O/C⟩^a^	⟨H/C⟩^a^	⟨OS_C_⟩^a^	remarks	
	VL*	POS: 0.42	0.87	–0.03	mainly dimeric and monomeric products (similar contributions)	
		NEG: 0.46	0.91	0.01		
	VL + ActSyr + SyrAld	POS: 0.50	0.97	0.04	more oxygenated and oxidized products than in VL* due to greater availability of oxidants promoting functionalization	
		NEG: 0.58	0.89	0.27		
	DMB + ActSyr + SyrAld	POS: 0.53	1	0.07	more oxygenated and oxidized products than in VL + ActSyr + SyrAld due to a greater and longer supply of oxidants initiating faster conversion of early generation products	
		NEG: 0.76	0.95	0.58		
	VL + ActSyr + SyrAld + GUA	POS: 0.65	0.87	0.43	more oxygenated and oxidized products than in mixed systems without GUA due to GUA transformation products; more evident change for VL + ActSyr + SyrAld + GUA possibly due to GUA oligomerization in DMB + ActSyr + SyrAld + GUA	
		NEG: 0.72	0.92	0.53		
	DMB + ActSyr + SyrAld + GUA	POS: 0.53	1	0.06		
		NEG: 0.81	0.91	0.71		

a The average elemental ratios (⟨O/C⟩ and ⟨H/C⟩) and ⟨OS_C_⟩ were based on the UHPLC-HESI-Orbitrap-MS results and estimated using the signal-weighted method.^92^

For VL*, monomer derivatives and dimers had nearly comparable signal contributions, indicating the similar significance of functionalization and oligomerization in this reaction system. Functionalization and oligomerization were also the major processes during the first 4 h of ActSyr and SyrAld (separately) photo-oxidation in ammonium nitrate or ammonium sulfate solutions.^59^ Our previous work on VL* showed that functionalization dominated over oligomerization at low VL concentration (0.01 mM), while oligomerization prevailed at high VL concentration (0.1 mM).^15^ Oligomer formation during aqueous-phase reactions of phenolic compounds requires the coupling of phenoxy radicals.^103,104^ Hence, high concentrations of phenolic compounds (e.g., ≥0.1 mM) can be expected to yield more oligomers due to higher phenoxy radical concentration, enhancing radical–radical polymerization.^104,105^ The different acquisition mechanisms and ionization methods used in our previous work (ESI-qToF-MS)^15^ and the present study (heated ESI-Orbitrap-MS) may have caused a discrepancy in the observed product distributions. For example, heated ESI provides an enhanced signal compared to unheated ESI, as the higher temperatures enable faster evaporation of droplets, thereby increasing the ionization efficiency of the analytes. Kourtchev et al. (2020)^106^ reported that during the analysis of atmospheric aerosol samples, the higher source temperature of heated ESI can potentially account for the higher recoveries of less volatile larger species compared to using unheated ESI. Based on this, the better ability of the heated ESI to ionize larger species can possibly account for the similar significance of functionalization and oligomerization for VL* in the current work.

Compared to single systems (VL* and DMB*), mixed systems (VL + ActSyr + SyrAld and DMB + ActSyr + SyrAld) have an increased availability of oxidizable substrates and oxidants (^3^C* and secondary oxidants). ^3^C*-derived secondary oxidants (e.g., ^•^OH and ^1^O_2_) can oxidize phenols or transient organic intermediates to generate functionalized species and highly oxidized ring-opening products.^9^ Also, as the solutions are air-saturated, the presence of O_2_ facilitates the generation of the peroxyl radical, an important intermediate in hydroxylation and ring-opening reactions.^11^ In VL + ActSyr + SyrAld, the greater availability of secondary oxidants facilitated functionalization and generation of more oxygenated aqSOA, as evidenced by the larger contribution of monomer derivatives and more products with H/C ≤ 1.0 and O/C ≥ 0.6 in comparison to VL*. The higher ⟨O/C⟩, ⟨H/C⟩, and ⟨OS_C_⟩ of VL + ActSyr + SyrAld aqSOA than VL* aqSOA denotes the prevalence of oxygenation and hydrogen incorporation reactions.^10^ Common formulas between VL + ActSyr + SyrAld and VL* correspond to 24 and 68% of the total number of their detected products, respectively (Figure 4a), suggesting that ActSyr and SyrAld oxidation products contributed significantly to VL + ActSyr + SyrAld aqSOA. This is aligned with ActSyr and SyrAld being oxidized faster than VL, as explained earlier. Also, there were more unique VL + ActSyr + SyrAld products within the LV-OOA range, consistent with more oxidized VL + ActSyr + SyrAld aqSOA than did VL* aqSOA (Figure 4b). As mentioned earlier, the DMB decay for DMB* was minimal, so MS analysis was not performed for this condition. Previously, we found that the stronger photosensitizing ability and photostability of DMB than VL enabled GUA oxidation by ^3^DMB* to occur faster and form products exhibiting greater light absorption than oxidation by ^3^VL*.^16^ In the present study, we further noted differences between aqSOA formations involving DMB and VL in mixed photosensitizer systems. For instance, monomer derivatives had a much higher signal contribution than dimers (by ∼11 times) for DMB + ActSyr + SyrAld than for VL + ActSyr + SyrAld (by ∼4 times). This difference can be attributed to DMB having greater photostability than the phenolic carbonyls (VL, ActSyr, and SyrAld), enabling the faster transformation of initially formed products in DMB + ActSyr + SyrAld due to its enhanced oxidant availability. Also, the more efficient SyrAld oxidation (1.3 times higher Φ_e_; *p* < 0.05) in DMB + ActSyr + SyrAld than in VL + ActSyr + SyrAld may have resulted in greater ^•^OH production.

In other words, the greater and longer supply of oxidants in DMB + ActSyr + SyrAld promoted the transformation of early generation aqSOA via fragmentation and ring-opening reactions, yielding less absorbing aqSOA (see later discussions).^10,17,107,108^ Indeed, the higher ⟨O/C⟩ and ⟨OS_C_⟩ in DMB + ActSyr + SyrAld aqSOA suggest more oxygenated and oxidized products than in VL + ActSyr + SyrAld aqSOA. Consistently, the higher concentration of total organic acid (e.g., formic acid) detected by IC analyses in DMB + ActSyr + SyrAld than in VL + ActSyr + SyrAld (Figure S6) indicates that oxidation-induced fragmentation was more important in the former. Likewise, the more extensive fragmentation during ActSyr and SyrAld (separately) photo-oxidation in ammonium nitrate than in ammonium sulfate solutions has been ascribed to the higher steady-state concentration of ^•^OH in ammonium nitrate solutions.^59^ GUA did not substantially affect the product distributions for both VL + ActSyr + SyrAld + GUA and DMB + ActSyr + SyrAld + GUA, in which monomer derivatives remain the highest signal contributors. This can be attributed to the higher reactivity of ActSyr and SyrAld relative to GUA, rendering them the primary oxidizable substrates in the reaction systems. Both VL + ActSyr + SyrAld + GUA and DMB + ActSyr + SyrAld + GUA yielded more oxygenated and oxidized aqSOA than mixed systems without GUA, as evident by increased ⟨O/C⟩ and ⟨OS_C_⟩. The greater ^3^C* availability from DMB compared to VL potentially initiated GUA oligomerization, leading to a smaller increase in ⟨O/C⟩ and ⟨OS_C_⟩ in DMB + ActSyr + SyrAld + GUA (vs DMB + ActSyr + SyrAld) than in VL + ActSyr + SyrAld + GUA (vs VL + ActSyr + SyrAld). Common formulas between VL + ActSyr + SyrAld + GUA and VL + ActSyr + SyrAld represented 61 and 77% of the total number of their detected products, respectively (Figure 4c). Compared to unique VL + ActSyr + SyrAld products, unique VL + ActSyr + SyrAld + GUA products exhibited broader ranges of O/C and H/C. Likewise, common formulas between DMB + ActSyr + SyrAld + GUA and DMB + ActSyr + SyrAld made up 67 and 71% of the total number of their detected products, respectively (Figure 4e). Notably, unique DMB + ActSyr + SyrAld + GUA products had a higher proportion of oligomers (45%) compared to DMB + ActSyr + SyrAld (21%), indicated by the cluster at O/C ≤ 0.5 and H/C ≤ 1.0. Overall, functionalization prevailed over oligomerization in mixed systems, while these processes had comparable significance in VL*. The greater oxidant availability in the mixed systems contributed to enhanced functionalization in VL + ActSyr + SyrAld and faster conversion of early generation aqSOA in DMB + ActSyr + SyrAld. Moreover, the formation of more oxygenated and oxidized aqSOA increased with the complexity of the reaction systems (e.g., VL* < VL + ActSyr + SyrAld < VL + ActSyr + SyrAld + GUA), which can be rationalized by the complex interactions among the photosensitizers in the mixed systems and their roles as oxidant sources and oxidizable substrates.

### Light Absorption

**Figure 5:**
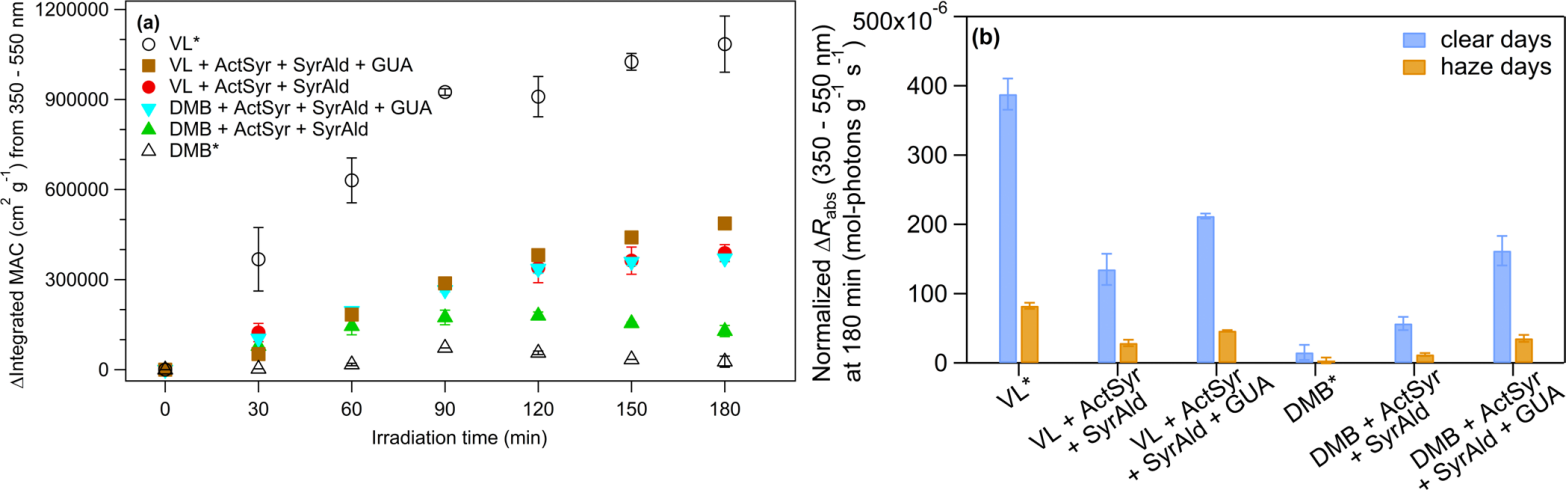
(a) Time profile of change in the integrated MAC and (b) change in the rate of sunlight absorption (Δ*R*_abs_) from 350 to 550 nm at 180 min normalized to the total initial reactant concentration in g cm^–3^ during typical clear and haze days in Beijing, China for VL*, VL + ActSyr + SyrAld, VL + ActSyr + SyrAld + GUA, DMB*, DMB + ActSyr + SyrAld, and DMB + ActSyr + SyrAld + GUA aqSOA. Error bars represent 1 standard deviation of triplicate experiments.

Visible light absorption enhancement during the aqueous-phase photo-oxidation of phenolic compounds and BB emissions has been associated with oligomerization and functionalization.^4,5,9,10,15−17,41,105,109^Figure 5 displays the time profile of change in the integrated mass absorption coefficient (MAC) and the change in the rate of sunlight absorption (Δ*R*_abs_)^10^ from 350 to 550 nm at 180 min normalized to the total initial reactant concentration in g cm^–3^ during typical clear and haze days in Beijing, China. Figure S7 shows the absorption spectra at 0 and 180 min of irradiation. Similar to aqueous-phase photo-oxidation of phenolic compounds,^4,5,10,15−17^ enhanced light absorption at wavelengths >350 nm was noted in all experiments, although DMB + ActSyr + SyrAld exhibited a decreasing trend at later reaction times. To identify potential BrC chromophores among the detected products, the DBE values were plotted against *n*_C_ (Figure S8; see Discussions in Section S9). VL* and VL + ActSyr + SyrAld aqSOA, VL + ActSyr + SyrAld and VL + ActSyr + SyrAld + GUA aqSOA, and DMB + ActSyr + SyrAld and DMB + ActSyr + SyrAld + GUA aqSOA hugely overlapped in the DBE vs *n*_C_ space, and most products were potential BrC chromophores. Generally, increased complexity of the reaction systems (e.g., VL* < VL + ActSyr + SyrAld < VL + ActSyr + SyrAld + GUA) led to more potential BrC chromophores.

VL* had significantly higher MAC enhancement and Δ*R*_abs_ values than the mixed systems. On a related note, field observations in a Beijing haze event reported that the oxidation of fossil-fuel primary organic aerosol aromatic species yielded aqSOA that is less light-absorbing relative to its primary precursor.^110^ The lower aqSOA light absorption in mixed systems possibly resulted from rivaling reactions involving the simultaneous formation of light-absorbing products and fragmentation processes producing less absorptive products. This can be attributed to the increased oxidant availability in mixed systems, contributing to the concurrent formation and decomposition of light-absorbing products. For DMB + ActSyr + SyrAld, the sustained and higher oxidant levels led to a declining trend of MAC enhancement toward the end of the experiment. As mentioned above, oxidation-induced fragmentation was more significant in DMB + ActSyr + SyrAld than in VL + ActSyr + SyrAld, as suggested by the higher concentration of total organic acid (e.g., formic acid) estimated via IC analyses. Similarly, it has been reported that the transformation of early generation products from photosensitized oxidation of guaiacyl acetone, a nonconjugated phenolic carbonyl, during prolonged photoaging may lead to chromophore degradation and aqSOA photobleaching.^10,11^ This was based on the correlation between reduced aqSOA light absorption and persistent increase of later-generation aqSOA consisting of a higher mass fraction of smaller fragmentation products relative to early generation aqSOA. Initial photoenhancement followed by photobleaching has been observed during the photochemical aging of model BB compounds, BB BrC proxy, and BrC from biomass pyrolysis.^10,107,108,111,112^ Zhong and Jang (2014) proposed that hydrogen peroxide (H_2_O_2_) from irradiation of nonphenolic carbonyls in the presence of phenols^26^ promoted photobleaching of wood smoke organic carbon. H_2_O_2_ photolysis yields ^•^OH, which can break down chromophores, contributing to decreased light absorption.^111^ Moreover, Hems et al. (2020) noted that the enhanced light absorption during UVB exposure and ^•^OH oxidation of GUA-containing wood smoke BrC can be correlated with the formation of aromatic dimers and functionalized products (only for ^•^OH oxidation).^109^ However, prolonged ^•^OH oxidation resulted in a net loss of absorbance comparable to that of the starting solution, which was linked to the loss of aromatic compounds and the breakdown to smaller molecules.

GUA transformation products prompted higher MAC enhancement and Δ*R*_abs_ from VL + ActSyr + SyrAld + GUA and DMB + ActSyr + SyrAld + GUA than from VL + ActSyr + SyrAld and DMB + ActSyr + SyrAld, respectively. Apart from reactions with ^3^C* and secondary oxidants, it was feasible that GUA also reacted with intermediates originating from aromatic carbonyls (e.g., aromatic radicals). The absorbance enhancement trends can reinforce the possibly more pronounced GUA oligomerization in DMB + ActSyr + SyrAld + GUA than in VL + ActSyr + SyrAld + GUA. Even though GUA led to slower decay and lower transformation efficiency for the aromatic carbonyl photosensitizers, as discussed earlier, its presence resulted in Δ*R*_abs_ greater by ∼4 times and 2 times for DMB + ActSyr + SyrAld + GUA and VL + ActSyr + SyrAld + GUA, respectively, compared to the corresponding mixed systems without GUA. Therefore, GUA oxidation products may be potentially important aqSOA light absorption contributors in DMB + ActSyr + SyrAld + GUA and VL + ActSyr + SyrAld + GUA, particularly for the former. Regardless, absorbance enhancement appeared to persist longer for VL + ActSyr + SyrAld + GUA, while that for DMB + ActSyr + SyrAld + GUA exhibited a plateau toward the later course of reactions, analogous to VL + ActSyr + SyrAld and DMB + ActSyr + SyrAld, respectively. These trends suggest a faster transformation into less-absorbing species for DMB + ActSyr + SyrAld + GUA early generation products than those from VL + ActSyr + SyrAld + GUA.

In our previous study on GUA oxidation by either ^3^VL* or ^3^DMB* only,^16^ continuous absorbance enhancement was noted, with ^3^DMB*-driven oxidation having higher Δ*R*_abs_, possibly due to greater oligomer contribution. Contrastingly, here, VL + ActSyr + SyrAld + GUA exhibited stronger absorbance enhancement than DMB + ActSyr + SyrAld + GUA. These opposing trends between GUA oxidation by ^3^VL* or ^3^DMB* only and VL + ActSyr + SyrAld + GUA and DMB + ActSyr + SyrAld + GUA were probably due to ActSyr and SyrAld serving as the main oxidizable substrates in mixed photosensitizer systems. As noted above, early generation DMB + ActSyr + SyrAld + GUA aqSOA have undergone faster conversion than those from VL + ActSyr + SyrAld + GUA, tapering light absorption. Thus, at prolonged irradiation, light absorption from ^3^DMB*-initiated GUA oxidation can be expected to show a decreasing trend earlier than that from ^3^VL*-driven oxidation. In brief, although mixed systems have more oxidizable substrates whose transformation products can enhance aqSOA light absorption, their stronger oxidative capacity can facilitate fragmentation, resulting in the formation of less-absorbing species. Consequently, VL + ActSyr + SyrAld aqSOA light absorption was lower than that of VL*. In addition, DMB + ActSyr + SyrAld aqSOA light absorption displayed a decreasing trend at the later irradiation stage.

## Atmospheric Implications

This study investigated aqSOA formation from the photochemical reactions of two mixed photosensitizer systems: VL + ActSyr + SyrAld and DMB + ActSyr + SyrAld. Moreover, photochemical reactions of VL and DMB in single photosensitizer systems (VL* and DMB*) were compared with those in VL + ActSyr + SyrAld and DMB + ActSyr + SyrAld. The behavior of mixed systems in the presence of GUA (VL + ActSyr + SyrAld + GUA and DMB + ActSyr + SyrAld + GUA), a noncarbonyl phenol, was also examined. We reached the following main findings, which highlight the dual role of organic photosensitizers in aqSOA and BrC formation as oxidant sources and oxidizable substrates. First, VL and DMB had shorter lifetimes in mixed systems (i.e., VL + ActSyr + SyrAld and DMB + ActSyr + SyrAld). For VL + ActSyr + SyrAld, this is ascribable to VL reacting with ^3^C* (and secondary oxidants) from the other photosensitizers. The increased DMB transformation in DMB + ActSyr + SyrAld could be due to coupling reactions with intermediates from the other photosensitizers or oxidation by ^•^OH generated in the system. Thus, VL and DMB would be enhanced aqSOA sources when present in mixed systems rather than in single systems. This also implies that aromatic carbonyl photosensitizers in mixed systems would have decreased availability for initiating other reactions, such as oxidizing noncarbonyl phenols and other organic compounds. Analyzing the contribution of photosensitized reactions to aqSOA formation requires the consideration of such conditions. Second, ^3^C*-derived secondary oxidants promoted functionalization in VL + ActSyr + SyrAld, while enhanced and prolonged oxidant supply accelerated early generation aqSOA transformation in DMB + ActSyr + SyrAld. The major aqSOA formation pathways for mixed and single systems were functionalization and oligomerization. These processes have comparable importance in VL*, but functionalization dominated in mixed systems. Consequently, more oxygenated and oxidized aqSOA was observed as the complexity of the reaction systems increased (e.g., VL* < VL + ActSyr + SyrAld < VL + ActSyr + SyrAld + GUA). Third, the lower aqSOA light absorption in mixed systems compared to single systems is attributable to more oxidizable substrates acting as aqSOA precursors, counteracted by the stronger oxidative capacity facilitating the formation of less-absorbing species. These opposing effects, which can influence the radiative forcing, were more evident for DMB + ActSyr + SyrAld, where aqSOA light absorption exhibited a declining pattern at later irradiation times. This emphasizes that the photosensitizing ability and photostability of individual aromatic carbonyl photosensitizers in mixed systems can affect aqSOA light absorption. Accordingly, the contribution of products from mixed systems to aqSOA light absorption would be lower than that of single systems.

Photosensitizers in mixed systems had longer lifetimes and decreased quantum yields in the presence of GUA. However, GUA had a minimal effect on the product distributions for VL + ActSyr + SyrAld + GUA and DMB + ActSyr + SyrAld + GUA, indicating that the photosensitized oxidation of GUA (and other noncarbonyl phenols) in mixed systems containing more reactive phenolic carbonyls (e.g., ActSyr and SyrAld) would be a less significant aqSOA source than in simpler binary systems (e.g., GUA oxidation by ^3^DMB* only). Nevertheless, GUA-derived products increased the aqSOA light absorption from mixed systems. In single systems, VL underwent rapid decay and showed significant photoenhancement, whereas DMB exhibited a slow decay and minimal photoenhancement. These indicate that direct photosensitized oxidation is important for VL* (and other phenolic carbonyl photosensitizers) but likely not for DMB*. These VL* findings, which are analogous to those of previous works,^4,15^ would be relevant for other phenolic carbonyl photosensitizers. On the other hand, we caution against generalizing the DMB* results to other nonphenolic carbonyl photosensitizers due to varying findings in earlier works. For instance, the photodegradation of other nonphenolic carbonyl photosensitizers, namely, 2-methoxybenzaldehyde,^26^ 3-methoxybenzaldehyde,^26^ and 9,10-anthraquinone^113^ in single systems, accompanied by the generation of H_2_O_2_, have been previously reported. Vione et al. (2006) attributed these observations to a reaction between the excited and ground-state photosensitizer, with the latter functioning as a substrate, resulting in photosensitizer transformation.^33^ Future research should explore the photochemical reactions of a greater diversity of nonphenolic carbonyl photosensitizers.

We note that the concentrations of compounds in this study represent typical levels in cloudwater/fogwater, but in aqueous aerosols, variations in reactant concentrations and identities, ionic strength, and pH can lead to substantially different reactions from those observed here. Understanding the effects of these environmental factors is imperative to enhancing our assessment of the contributions of photosensitized processes to aqSOA formation. Thus, photosensitization studies conducted under a wider range of atmospherically relevant conditions are warranted. In addition, other oxidants in cloud/fogwater, such as ^•^OH, may also affect the reactions discussed. Therefore, photosensitized aqSOA formation studies should consider the simultaneous generation of ^3^C* from organic photosensitizers and their oxidation. Further, this study focused on certain aromatic carbonyls, but there are other less-studied organic photosensitizers with greater ^3^C* formation efficiency (e.g., benzophenone and xanthone).^30^ Future studies should also explore more multicomponent mixtures involving other organic compounds (e.g., aliphatic polyols and carboxylic acids) and inorganic compounds (e.g., ammonium nitrate,^15,16,114^ ammonium chloride,^115^ and sulfur dioxide^38,116,117^), especially those with high atmospheric abundance, for a better understanding of atmospheric photosensitization.

## Supporting Information Available

Details of the analytical procedures; calculations of effective quantum yields and estimated atmospheric lifetimes; discussions on the major products detected, van Krevelen diagrams and OS_C_ vs *n*_C_ plots, and DBE values vs *n*_C_ plots (PDF)
