# Supplementary material for: Aqueous-Phase Photoreactions of Mixed Aromatic Carbonyl Photosensitizers Yield More Oxygenated, Oxidized, and less Light-Absorbing Secondary Organic Aerosol (SOA) than Single Systems
Source: Environ Sci Technol. 2024 Apr 23;58(18):7924–36. doi: 10.1021/acs.est.3c10199 (PMC11080053; doi:10.1021/acs.est.3c10199)
Supplement: Supplementary file 1 — es3c10199_si_001.pdf [file es3c10199_si_001.pdf]

Supplementary Information for

Aqueous-phase photoreactions of mixed aromatic carbonyl photosensitizers yield more oxygenated, oxidized, and less light-absorbing secondary organic aerosol (SOA) than single systems

Beatrix Rosette Go Mabato[1], Yong Jie Li[2], Dan Dan Huang[3], Chak K. Chan[1,4*]

[1]School of Energy and Environment, City University of Hong Kong, Tat Chee Avenue, Kowloon 999077, Hong Kong SAR, China

[2]Department of Civil and Environmental Engineering, and Centre for Regional Ocean, Faculty of Science and Technology, University of Macau, Macau 999078, China

[3]Shanghai Academy of Environmental Sciences, Shanghai 200233, China

[4]Division of Physical Sciences and Engineering, King Abdullah University of Science and Technology (KAUST), Thuwal, Jeddah 23955-6900, Kingdom of Saudi Arabia

Summary: 25 pages, 8 figures, 6 tables.

Text S1. UHPLC-PDA analyses.

The concentrations of aromatic carbonyl photosensitizers (vanillin [VL], acetosyringone [ActSyr], syringaldehyde [SyrAld], and 3,4-dimethoxybenzaldehyde [DMB]) and guaiacol (GUA) were measured using an ultra-high performance liquid chromatography system (UHPLC, Waters Acquity H-Class, Waters, Milford, USA) coupled to a photodiode array (PDA) detector (Waters, Milford, USA). Prior to UHPLC analysis, the samples were filtered via a 0.2 μm Chromafil® Xtra PTFE filter (Macherey-Nagel GmbH & Co. KG, Germany). The separation of products was enabled by an Acquity HSS T3 column (1.8 μm, 2.1 mm × 100 mm; Waters Corp.). The column oven was held at 30 °C, and the autosampler was cooled at 4 °C. The injection volume used was 5 μL. The binary mobile phase consisted of water (A) and acetonitrile (B). The gradient elution was performed at a flow rate of 0.2 mL min^{-1}: 0–1 min, 10% eluent B; 1–25 min, linear increase to 90% eluent B; 25–29.9 min, hold 90% eluent B; 29.9–30 min, decrease to 10% eluent B; 30–35 min, then re-equilibrate at 10% eluent B for 5 min. The channels with UV absorption at 300, 274, 295, 305, and 274 nm were used to analyze VL, DMB, ActSyr, SyrAld, and GUA respectively.

Text S2. UHPLC-HESI-Orbitrap-MS analyses.

The detection of reaction products was conducted with a Thermo Orbitrap Fusion Lumos Mass Spectrometry (Thermo Fisher Scientific, Waltham, MA, USA) connected to a Thermo Scientific UltiMate 3000 UHPLC system (Thermo Fisher Scientific, Waltham, MA, USA) via heated electrospray ionization (HESI) interface (UHPLC-HESI-Orbitrap-MS). The mobile phases used were 0.1% (v/v) formic acid (in milli-Q water) (A) and acetonitrile (B). The UHPLC-HESI-Orbitrap-MS system settings (e.g., column, gradient, oven temperature) were identical to those used in the UHPLC analysis (Section S1). The HESI-MS spectra were acquired in both positive and negative ion modes. The HESI parameters were the following: Spray voltage, 2500 V for both positive and negative HESI; sheath gas, 35 arbitrary units;

nebulizer auxiliary gas, 10 arbitrary units; sweep gas, 3 arbitrary units. General instrumental parameters were set as follows: ion transfer tube temperature, 320 °C; vaporizer temperature, 350 °C. The mass range for full scan MS was set at 50-1000 m/z with a mass resolution of 60,000 at 200 m/z. The automatic gain control (AGC) target was 4.0×10^5 with a maximum injection time of 50 ms. Peak picking and alignment were performed using Progenesis QI (version 2.4; Nonlinear Dynamics). The majority of the peaks detected in the blank (~99% for all experiments) were disregarded in the samples apart for peaks with a minimum of 2.5 times greater intensity in the sample spectrum than in the blank.[1] Moreover, a peak was treated as a product if the difference in the peak area between the samples before and after irradiation is ≥10 times. In this study, two independently prepared samples for each reaction system were subjected to UHPLC-HESI-Orbitrap-MS analysis. Only the consistently observed peaks in both sets of samples were retained. The formula assignments were accomplished using the MIDAS molecular formula calculator (http://magnet.fsu.edu/~midas/) with the following constraints: C ≤100, H ≤150, and O ≤30, and mass error of 10 ppm. In addition, the ChemSpider database (Royal Society of Chemistry) was queried to provide valid molecules that may assist in suggesting product structures. In general, the proposed structures here are based on molecular formulas, double bond equivalent (DBE) values, and structural and mechanistic information derived from previous related works on aromatic carbonyls and GUA.[2–11] For clarity, the formulas presented in this study correspond to neutral analytes (e.g., with H^+ or NH_4^+ removed from the ion formula). The following equations were used to calculate the DBE values[12] and carbon oxidation state (OS_C)[13–15] of the neutral formulas:

$$DBE = C - H/2 + 1 \quad \text{(Eq. S1)}$$

$$OS_C = 2 \times O/C - H/C \quad \text{(Eq. S2)}$$

where C, H, and O correspond to the number of carbon, hydrogen, and oxygen atoms in the neutral formula.

Additionally, the signal-weighted method[16] was used to determine the average oxygen to carbon (O/C) ratios, ⟨O/C⟩: ($\langle \mathrm{O/C} \rangle = \sum_i (\mathrm{abundance_i}) O_i / \sum_i (\mathrm{abundance}_i) C_i$) and average hydrogen to carbon (H/C) ratios, ⟨H/C⟩: ($\langle \mathrm{H/C} \rangle = \sum_i (\mathrm{abundance}_i) H_i / \sum_i (\mathrm{abundance_i}) C_i$) after the reactions. The average OS_C, $\langle OS_C \rangle$ was also quantified as follows:

$$\langle OS_C \rangle = 2 \times \langle O/C \rangle - \langle H/C \rangle \quad \text{(Eq. S3)}$$

Text S3. UV-Vis spectrophotometric analyses.

The absorbance changes were measured using a UV-Vis spectrophotometer (UV-3600, Shimadzu Corp., Japan). The absorbance values from 200 to 700 nm were promptly recorded after sample collection, with measurements conducted in triplicate. The absorbance data was converted to mass absorption coefficients (MAC, cm^2 g^{-1}) as follows:[17,18]

$$\mathrm{MAC}\ (\lambda) = \frac{\mathrm{Abs}_e\ (\lambda)}{b C_{\mathrm{mass}}} \quad \text{(Eq. S4)}$$

where Abs_e is base-*e* absorbance at wavelength λ, b is path length in cm, and C_{mass} is the total initial reactant concentration in g cm^{-3}.

Additionally, the change in the integrated MAC from 350 to 550 nm represented absorbance enhancements.

Text S4. IC analyses of small organic acids.

The concentrations of small organic acids were quantified using an ion chromatography system (IC, Dionex ICS-1100, Sunnyvale, CA) equipped with a Dionex AS-DV autosampler (Sunnyvale, CA). The separation was enabled using an IonPac™ AS11 column (4 × 250 mm) with an IonPac™ AG11 guard column (4 × 50 mm). Elution was performed isocratically at a flow rate of 1.0 mL min^{-1}, employing 12 mM sodium hydroxide (NaOH) as the eluent. The total run time was established to be 10 minutes. The standard solutions (1–50 μM) of formic, succinic, and oxalic acid were analyzed three times alongside the samples and a water blank. The retention times of formic, succinic, and oxalic acid were 1.9 min, 3.7 min, and 5.9 min, respectively.

Text S5. Calculation of effective quantum yields.

The effective quantum yields (Φ_e) were calculated using the following equation:[19–21]

$$\Phi_e = \frac{k'[C]}{\sum f_i(\lambda) I_0(\lambda)(1-10^{-\varepsilon(\lambda)\times[C]\times l})\Delta\lambda} \cdot f_{2NB,corr} \quad \text{(Eq. S5)}$$

where *k'* is pseudo-first-order decay rate constant (s^{-1}); [C] is concentration (M) of the aromatic carbonyl photosensitizer or GUA; and $f_i(\lambda)$ is the relative light absorption, which is the fraction of light absorption by an aromatic carbonyl photosensitizer at a specific wavelength (see Eqs. S6-S8). For single photosensitizer systems (i.e., VL* and DMB*), $f_i(\lambda) = 1$, whereas $f_i(\lambda)$ was employed to measure the light competition in mixed photosensitizer systems (e.g., VL + ActSyr + SyrAld). $I_0(\lambda)$ is the volume-normalized light intensity (Einstein L^{-1} s^{-1} nm^{-1}) of the lamp; $\varepsilon(\lambda)$ is the base-10 molar absorptivity (M^{-1} cm^{-1}); *l* is the pathlength of the illumination cell (cm); and $\Delta\lambda$ is the interval of wavelength (nm). A factor ($f_{2NB,corr}$) derived from the photolysis of 2-nitrobenzaldehyde (2NB), a chemical actinometer, was used to correct the Φ_e values, to scale the light intensity to that estimated from the chemical actinometry (see Wang et al., 2023 for more information).[21]

For mixed photosensitizer systems, the Φ_e values were estimated as follows:

$$\Phi_{e,1} = \frac{k'_1[C_1]}{\sum I_0(\lambda)\times(1-10^{-[\varepsilon_1(\lambda)C_1+\varepsilon_2(\lambda)C_2+\varepsilon_3(\lambda)C_3]\times l})\times f_1(\lambda)\times\Delta\lambda}, \quad f_1(\lambda) = \frac{\varepsilon_1(\lambda)C_1}{\varepsilon_1(\lambda)C_1+\varepsilon_2(\lambda)C_2+\varepsilon_3(\lambda)C_3} \quad \text{(Eq. S6)}$$

$$\Phi_{e,2} = \frac{k'_2[C_2]}{\sum I_0(\lambda)\times(1-10^{-[\varepsilon_1(\lambda)C_1+\varepsilon_2(\lambda)C_2+\varepsilon_3(\lambda)C_3]\times l})\times f_2(\lambda)\times\Delta\lambda}, \quad f_2(\lambda) = \frac{\varepsilon_2(\lambda)C_2}{\varepsilon_1(\lambda)C_1+\varepsilon_2(\lambda)C_2+\varepsilon_3(\lambda)C_3} \quad \text{(Eq. S7)}$$

$$\Phi_{e,3} = \frac{k'_3[C_3]}{\sum I_0(\lambda)\times(1-10^{-[\varepsilon_1(\lambda)C_1+\varepsilon_2(\lambda)C_2+\varepsilon_3(\lambda)C_3]\times l})\times f_3(\lambda)\times\Delta\lambda}, \quad f_3(\lambda) = \frac{\varepsilon_3(\lambda)C_3}{\varepsilon_1(\lambda)C_1+\varepsilon_2(\lambda)C_2+\varepsilon_3(\lambda)C_3} \quad \text{(Eq. S8)}$$

where $[C_1]$, $[C_2]$, and $[C_3]$ are the concentrations (M) of aromatic carbonyl photosensitizer 1, 2, and 3, respectively; ε_1, ε_2, and ε_3 are the base-10 molar absorptivities (M^{-1} cm^{-1}) of aromatic carbonyl photosensitizer 1, 2, and 3, respectively; and $f_1(\lambda)$, $f_2(\lambda)$, and $f_3(\lambda)$ are the fraction of light absorption by aromatic carbonyl photosensitizer 1, 2, and 3, respectively.

Text S6. Calculation of estimated atmospheric lifetimes.

The effective quantum yields (Φ_e) were used to estimate the atmospheric lifetimes (τ) of the aromatic carbonyl photosensitizers. The photolysis rate constant (j, s^{-1}) for dilute cloud and fog water can be determined as follows:[21,22]

$$j = 3.82 \times 10^{-21} \phi \sum F(\lambda)\varepsilon(\lambda)\Delta\lambda \qquad \text{(Eq. S9)}$$

where 3.82×10^{-21} is used to convert the absorption coefficient from the base-10 form (typically in the unit of M^{-1} cm^{-1}) to the base-*e* form (typically in the unit of cm^2 $molecule^{-1}$); Φ is the intrinsic quantum yield of aromatic carbonyl decay (dimensionless); and $F(\lambda)$ is wavelength-dependent solar flux (photon cm^{-2} s^{-1} nm^{-1}).

The atmospheric lifetime (τ) of the aromatic carbonyl photosensitizers during irradiation is determined by taking the reciprocal of the photolysis rate constant (j):

$$\tau = \frac{1}{j} \qquad \text{(Eq. S10)}$$

Text S7. Discussions on the major products detected from VL*, VL + ActSyr + SyrAld, DMB + ActSyr + SyrAld, VL + ActSyr + SyrAld + GUA, and DMB + ActSyr + SyrAld + GUA.

VL + ActSyr + SyrAld had more monomer derivatives as major products, while VL* had more high-relative-abundance dimeric products. Several common major products observed between VL + ActSyr + SyrAld and VL* include $C_8H_8O_4$ (a demethylated SyrAld or a hydroxylated VL, no. 1; Table S2) and $C_{16}H_{14}O_6$ (potentially a VL dimer, no. 3; Table S2). Both $C_8H_8O_4$ and $C_{16}H_{14}O_6$ have also been detected from VL* at 0.1 mM VL in our previous study.[10] In VL + ActSyr + SyrAld, ring-opening pathways likely contributed to the greater relative abundance of functionalized monomers. For instance, high-relative-abundance VL + ActSyr + SyrAld products include $C_{13}H_{14}O_8$ (no. 7; Table S2), a highly functionalized

monomer with a substituent corresponding to a highly oxygenated moiety of fragmentation reactions and $C_8H_4O_5$ (no. 14; Table S2) which potentially possess a furanone group.

Among the common species observed between SyrAld photo-oxidation in ammonium nitrate or ammonium sulfate solutions[23] and mixed photosensitizer systems studied here were $C_8H_8O_4$ (no. 1; Table S2), $C_7H_6O_5$ (no. 11; Table S2), and $C_7H_6O_4$ (no. 35; Table S4). $C_8H_8O_4$, the major product of both VL + ActSyr + SyrAld and DMB + ActSyr + SyrAld, was likely a demethylated SyrAld. In addition, $C_7H_6O_5$ and $C_7H_6O_4$ were potentially formed via oxidation of aldehyde group of SyrAld, demethylation, and demethoxylation.

More than half of the high-relative-abundance VL + ActSyr + SyrAld + GUA and DMB + ActSyr + SyrAld + GUA products were also detected from the corresponding mixed photosensitizer systems without GUA (i.e., VL + ActSyr + SyrAld and DMB + ActSyr + SyrAld). Likewise, the major products from DMB + ActSyr + SyrAld were mostly detected from VL + ActSyr + SyrAld as well (Tables S2 and S5). These observations were in agreement with ActSyr and SyrAld having higher reactivity than GUA and being the main oxidizable substrates in all mixed photosensitizer systems examined. The major products that possibly originated from GUA include $C_7H_8O_4$ (no. 32; Table S4), which could be a hydroxylated GUA, and $C_{13}H_{12}O_4$ (no. 48; Table S6), which could be a GUA dimer. $C_7H_8O_4$ was among the high-relative-abundance VL + ActSyr + SyrAld + GUA and DMB + ActSyr + SyrAld + GUA products, while $C_{13}H_{12}O_4$ was noted only from DMB + ActSyr + SyrAld + GUA major products. Both $C_7H_8O_4$ and $C_{13}H_{12}O_4$ have been reported from GUA oxidation by ^{3}DMB* or ^{3}VL* only.[4,5,10,11] Notable among the unique high-relative-abundance VL + ActSyr + SyrAld + GUA products were $C_{14}H_{16}O_8$ and $C_8H_8O_7$ (nos. 31 and 36, respectively; Table S4), which are highly oxygenated, functionalized monomers.

Text S8. Discussions on the van Krevelen diagrams and OS_C vs. n_C plots for VL*, VL + ActSyr + SyrAld, DMB + ActSyr + SyrAld, VL + ActSyr + SyrAld + GUA, and DMB + ActSyr + SyrAld + GUA aqSOA.

High-relative-abundance VL* products had O/C between 0.30-0.60 and H/C between 0.75-1, representing VL dimers ($C_{16}H_{14}O_6$, no. 3; Table S2) and functionalized VL (e.g., $C_8H_8O_5$, no. 12; Table S2) (Figure S4a). Similarly, $C_{16}H_{14}O_6$ was also among the high-relative-abundance VL + ActSyr + SyrAld species, but functionalized monomers with higher O/C (0.40-0.70) and H/C (0.86-1) (e.g., $C_8H_8O_4$, $C_7H_6O_3$, and $C_7H_6O_5$, nos. 1, 10, and 11, respectively; Table S2) had higher relative abundance in VL + ActSyr + SyrAld than in VL* (Figure S4b,c). The van Krevelen diagrams for VL + ActSyr + SyrAld + GUA and DMB + ActSyr + SyrAld + GUA largely resembled those of mixed systems without GUA (Figure S4b–i), yet noticeable differences were observed. Compared to VL + ActSyr + SyrAld, there were high-relative-abundance species with high O/C (≥0.5) and H/C (up to 1.2) in VL + ActSyr + SyrAld + GUA, corresponding to highly oxygenated, functionalized monomers (e.g., $C_{11}H_{12}O_7$, $C_{12}H_{12}O_6$, and $C_8H_8O_7$, nos. 29, 30, and 36, respectively; Table S4). In DMB + ActSyr + SyrAld + GUA, there was a cluster of species at O/C ~0.5 and H/C ~1, ascribable to dimeric species such as $C_{13}H_{12}O_4$ (no. 48; Table S6).

Among the high-relative-abundance VL* and VL + ActSyr + SyrAld species were $C_{16}H_{14}O_6$ (no. 3; Table S2) and $C_8H_8O_5$ (no. 12; Table S2), which can be classified as semivolatile oxygenated organic aerosol (SV-OOA) and low-volatility oxygenated organic aerosol (LV-OOA),[13] respectively (Figure S5a–c). However, compared to VL*, VL + ActSyr + SyrAld had more unique high-relative-abundance products characterized as LV-OOA (e.g., $C_7H_4O_3$ and $C_5H_8O_5$, nos. 2 and 5, respectively; Table S2). Relative to mixed systems without GUA, VL + ActSyr + SyrAld + GUA and DMB + ActSyr + SyrAld + GUA had more high-relative-abundance species corresponding to LV-OOA (Figure S5b–i), in accordance with

more oxidized products noted in mixed systems with GUA. Examples of high-relative-abundance-species associated with LV-OOA for VL + ActSyr + SyrAld + GUA and DMB + ActSyr + SyrAld + GUA were $C_8H_8O_7$ (no. 36; Table S4) and $C_{12}H_{10}O_7$ (no. 44; Table S6), respectively.

Section S9. Discussions on the DBE values vs. n_C plots for VL*, VL + ActSyr + SyrAld, DMB + ActSyr + SyrAld, VL + ActSyr + SyrAld + GUA, and DMB + ActSyr + SyrAld + GUA aqSOA.

The plot of the DBE values vs. n_C can be used to distinguish potential brown (BrC) chromophores among the generated aqSOA.[24] These plots are shown in Figure S8, with the DBE reference values of fullerene-like hydrocarbons,[25] cata-condensed polycyclic aromatic hydrocarbons (PAHs),[26] and linear conjugated polyenes with a general formula C_xH_{x+2}. Since BrC light absorption necessitates continuous conjugation across a substantial portion of the molecular structures, species with DBE / n_C ratios higher than that of linear conjugated polyenes (shaded area in Figure S8) are potential BrC compounds.[24] In other words, species within the shaded area have sufficient conjugation for visible light absorption and are potential BrC chromophores.

High-relative-abundance potential BrC chromophores from VL + ActSyr + SyrAld and VL* include a dimeric species ($C_{16}H_{14}O_6$; 10 DBE, no. 3; Table S2) and functionalized monomers ($C_7H_6O_3$ and $C_8H_8O_5$; 5 DBE, nos. 10 and 12, respectively; Table S2). Additionally, VL + ActSyr + SyrAld had more unique functionalized monomers (e.g., $n_C \geq 7$ and DBE ≥ 5) including $C_{11}H_{10}O_5$, $C_{13}H_{14}O_8$, $C_8H_4O_5$, and $C_{10}H_{12}O_6$ (nos. 4, 7, 14, and 15, respectively; Table S2) that are potential BrC chromophores than VL*, which was more apparent in the positive ion mode data (Figure S8a,b). A greater number of potential BrC chromophores with higher n_C and DBE (both ≥ 10) were observed from mixed systems in the presence of GUA. In particular, potential BrC chromophores corresponding to highly functionalized monomers

(e.g., n_C = 10-14 and 6-7 DBE such as $C_{11}H_{12}O_7$; no. 29; Table S4) had higher relative abundance in VL + ActSyr + SyrAld + GUA than in VL + ActSyr + SyrAld (Figure S8c,d). DMB + ActSyr + SyrAld and DMB + ActSyr + SyrAld + GUA shared similar high-relative-abundance functionalized monomers with n_C = 6-9 and 4-6 DBE (e.g., $C_8H_8O_4$ and $C_7H_8O_3$, nos. 1 and 8, respectively; Table S2). In DMB + ActSyr + SyrAld + GUA, there was also a cluster of species with n_C = 13-15 and 9 DBE assignable to dimeric compounds (Figure S8e,f).

Table S1. List of experiments performed for single and mixed photosensitizer systems.

Reaction conditions	Descriptions
VL*	Single phenolic carbonyl photosensitizer system
DMB*	Single non-phenolic carbonyl photosensitizer system
VL + ActSyr + SyrAld	Mixed phenolic carbonyl photosensitizer system
DMB + ActSyr + SyrAld	Mixed non-phenolic and phenolic carbonyl photosensitizer system
VL + ActSyr + SyrAld + GUA	Mixed phenolic carbonyl photosensitizer system in the presence of a non-carbonyl phenol
DMB + ActSyr + SyrAld + GUA	Mixed non-phenolic and phenolic carbonyl photosensitizer system in the presence of a non-carbonyl phenol

Table S2. Possible structures of major VL + ActSyr + SyrAld products detected using UHPLC-HESI-Orbitrap-MS operated in positive (POS) and negative (NEG) ion modes.

No.	VL + ActSyr + SyrAld POS Molecular formula and exact mass	DBE	Possible structure	No.	VL + ActSyr + SyrAld NEG Molecular formula and exact mass	DBE	Possible structure
1	$C_8H_8O_4$ (168.0423)	5	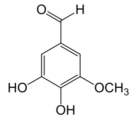	$C_{16}H_{14}O_6$ (302.0790) (No. 3; VL + ActSyr + SyrAld POS)			
2	$C_7H_4O_3$ (136.0160)	6	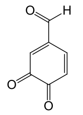	$C_7H_6O_3$ (138.0317) (No. 10; VL + ActSyr + SyrAld POS)			
3	$C_{16}H_{14}O_6$ (302.0790)	10	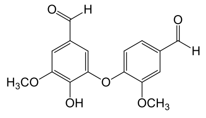	11	$C_7H_6O_5$ (170.0215)	5	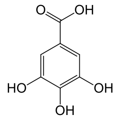
4	$C_{11}H_{10}O_5$ (222.0528)	7	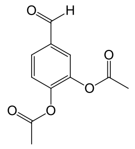	12	$C_8H_8O_5$ (184.0372)	5	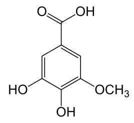
5	$C_5H_8O_5$ (148.0372)	2	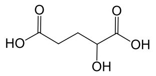	13	$C_7H_4O_6$ (184.0008)	6	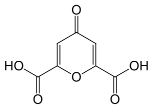
6	$C_8H_{10}O_4$ (170.0579)	4	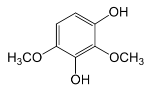	14	$C_8H_4O_5$ (180.0059)	7	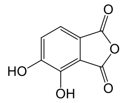
7	$C_{13}H_{14}O_8$ (298.0689)	7	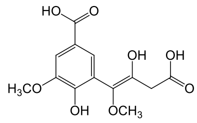	15	$C_{10}H_{12}O_6$ (228.0634)	5	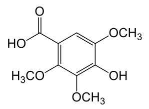
8	$C_7H_8O_3$ (140.0473)	4	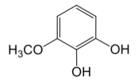	$C_8H_8O_4$ (168.0423) (No. 1; VL + ActSyr + SyrAld POS)			
9	$C_8H_6O_4$ (166.0266)	6	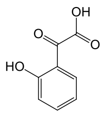	16	$C_7H_6O_7$ (202.0113)	5	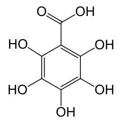
10	$C_7H_6O_3$ (138.0317)	5	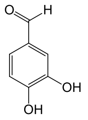	17	$C_9H_8O_7$ (228.0270)	6	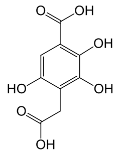

Table S3. Possible structures of major VL* products detected using UHPLC-HESI-Orbitrap-MS operated in positive (POS) and negative (NEG) ion modes.

No.	VL* POS Molecular formula and exact mass	DBE	Possible structure	No.	VL* NEG Molecular formula and exact mass	DBE	Possible structure
$C_{16}H_{14}O_6$ (302.0790) (No. 3; VL + ActSyr + SyrAld POS, Table S2)				$C_{16}H_{14}O_6$ (302.0790) (No. 3; VL + ActSyr + SyrAld POS, Table S2)			
$C_8H_6O_4$ (166.0266) (No. 9; VL + ActSyr + SyrAld POS, Table S2)				$C_8H_8O_5$ (184.0372) (No. 12; VL + ActSyr + SyrAld NEG, Table S2)			
18	$C_{11}H_{10}O_6$ (238.0477)	7	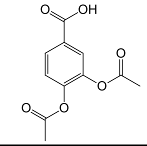	$C_8H_8O_4$ (168.0423) (No. 1; VL + ActSyr + SyrAld POS, Table S2)			
19	$C_{10}H_8O_4$ (192.0423)	7	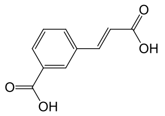	$C_6H_4O_3$ (124.0160) (No. 21; VL* POS)			
20	$C_{10}H_{10}O_5$ (210.0528)	6	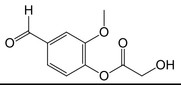	24	$C_5H_6O_5$ (146.0215)	3	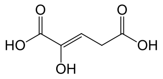
$C_7H_6O_3$ (138.0317) (No. 10; VL + ActSyr + SyrAld POS, Table S2)				$C_7H_6O_5$ (170.0215) (No. 11; VL + ActSyr + SyrAld NEG, Table S2)			
21	$C_6H_4O_3$ (124.0160)	5	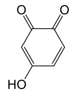	25	$C_8H_6O_6$ (198.0164)	6	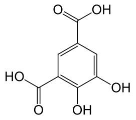
22	$C_7H_8O_5$ (172.0372)	4	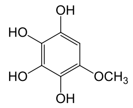	26	$C_5H_4O_3$ (112.0160)	4	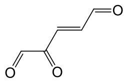
23	$C_6H_6O_6$ (174.0164)	4	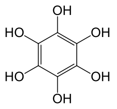	27	$C_{14}H_{14}O_6$ (278.0790)	8	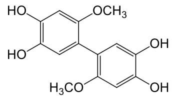
$C_7H_8O_3$ (140.0473) (No. 8; VL + ActSyr + SyrAld POS, Table S2)				28	$C_{16}H_{14}O_7$ (318.0740)	10	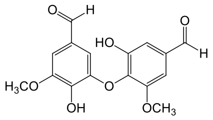

Table S4. Possible structures of major VL + ActSyr + SyrAld + GUA products detected using UHPLC-HESI-Orbitrap-MS operated in positive (POS) and negative (NEG) ion modes.

No.	VL + ActSyr + SyrAld + GUA POS Molecular formula and exact mass	DBE	Possible structure	No.	VL + ActSyr + SyrAld + GUA NEG Molecular formula and exact mass	DBE	Possible structure
$C_8H_8O_4$ (168.0423) (No. 1; VL + ActSyr + SyrAld POS, Table S2)				$C_7H_6O_5$ (170.0215) (No. 11; VL + ActSyr + SyrAld NEG, Table S2)			
29	$C_{11}H_{12}O_7$ (256.0583)	6	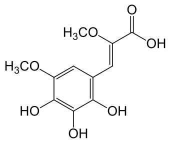	$C_7H_6O_7$ (202.0113) (No. 16; VL + ActSyr + SyrAld NEG, Table S2)			
30	$C_{12}H_{12}O_6$ (252.0634)	7	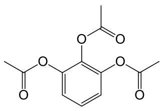	$C_{16}H_{14}O_6$ (302.0790) (No. 3; VL + ActSyr + SyrAld POS Table S2)			
31	$C_{14}H_{16}O_8$ (312.0845)	7	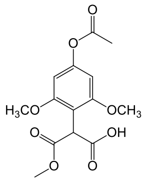	$C_8H_8O_4$ (168.0423) (No. 1; VL + ActSyr + SyrAld POS Table S2)			
$C_{11}H_{10}O_5$ (222.0528) (No. 4; VL + ActSyr + SyrAld POS, Table S2)				$C_8H_8O_5$ (184.0372) (No. 12; VL + ActSyr + SyrAld NEG, Table S2)			
$C_8H_6O_4$ (166.0266) (No. 9; VL + ActSyr + SyrAld POS, Table S2)				$C_7H_4O_6$ (184.0008) (No. 13; VL + ActSyr + SyrAld NEG, Table S2)			
$C_8H_6O_6$ (198.0164) (No. 25; VL* NEG, Table S3)				34	$C_{15}H_{14}O_5$ (274.0841)	9	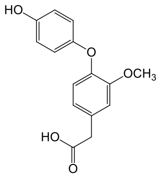
32	$C_7H_8O_4$ (156.0423)	4	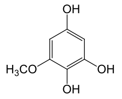	$C_9H_8O_7$ (228.0270) (No. 17; VL + ActSyr + SyrAld NEG, Table S2)			
33	$C_8H_{10}O_5$ (186.0528)	4	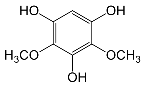	35	$C_7H_6O_4$ (154.0266)	5	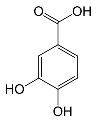
$C_7H_6O_5$ (170.0215) (No. 11; VL + ActSyr + SyrAld NEG, Table S2)				36	$C_8H_8O_7$ (216.0270)	5	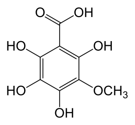

Table S5. Possible structures of major DMB + ActSyr + SyrAld products detected using UHPLC-HESI-Orbitrap-MS operated in positive (POS) and negative (NEG) ion modes.

No.	DMB + ActSyr + SyrAld POS Molecular formula and exact mass	DBE	Possible structure	No.	DMB + ActSyr + SyrAld NEG Molecular formula and exact mass	DBE	Possible structure
$C_8H_8O_4$ (168.0423) (No. 1; VL + ActSyr + SyrAld POS, Table S2)				$C_7H_6O_5$ (170.0215) (No. 11; VL + ActSyr + SyrAld NEG, Table S2)			
$C_{13}H_{14}O_8$ (298.0689) (No. 7; VL + ActSyr + SyrAld POS, Table S2)				$C_9H_8O_7$ (228.0270) (No. 17; VL + ActSyr + SyrAld NEG, Table S2)			
$C_7H_8O_3$ (140.0473) (No. 8; VL + ActSyr + SyrAld POS, Table S2)				$C_7H_6O_7$ (202.0113) (No. 16; VL + ActSyr + SyrAld NEG, Table S2)			
$C_8H_{10}O_5$ (186.0528) (No. 33; VL + ActSyr + SyrAld + GUA POS, Table S4)				$C_8H_8O_7$ (216.0270) (No. 36; VL + ActSyr + SyrAld + GUA NEG, Table S4)			
$C_7H_6O_4$ (154.0266) (No. 35; VL + ActSyr + SyrAld + GUA NEG, Table S4)				$C_{10}H_{12}O_6$ (228.0634) (No. 15; VL + ActSyr + SyrAld NEG, Table S2)			
$C_8H_8O_5$ (184.0372) (No. 12; VL + ActSyr + SyrAld NEG, Table S2)				39	$C_{14}H_{14}O_9$ (326.0638)	8	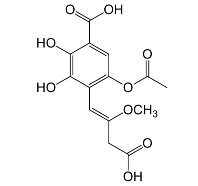
37	$C_9H_8O_5$ (196.0372)	6	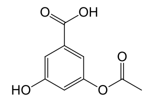	40	$C_9H_{10}O_7$ (230.0427)	5	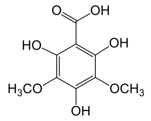
$C_{12}H_{12}O_6$ (252.0634) (No. 30; VL + ActSyr + SyrAld + GUA POS, Table S4)				41	$C_7H_6O_6$ (186.0164)	5	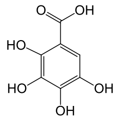
38	$C_{14}H_{16}O_6$ (280.0947)	7	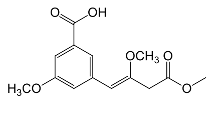	42	$C_8H_{10}O_7$ (218.0427)	4	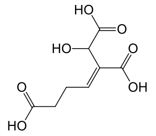
$C_{10}H_{12}O_6$ (228.0634) (No. 15; VL + ActSyr + SyrAld NEG, Table S2)				43	$C_9H_{10}O_6$ (214.0477)	5	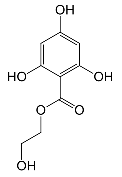

Table S6. Possible structures of major DMB + ActSyr + SyrAld + GUA products detected using UHPLC-HESI-Orbitrap-MS operated in positive (POS) and negative (NEG) ion modes.

No.	DMB + ActSyr + SyrAld + GUA POS Molecular formula and exact mass	DBE	Possible structure	No.	DMB + ActSyr + SyrAld + GUA NEG Molecular formula and exact mass	DBE	Possible structure
$C_8H_8O_4$ (168.0423) (No. 1; VL + ActSyr + SyrAld POS, Table S2)				$C_{10}H_{12}O_6$ (228.0634) (No. 15; VL + ActSyr + SyrAld NEG, Table S2)			
$C_7H_8O_3$ (140.0473) (No. 8; VL + ActSyr + SyrAld POS, Table S2)				$C_7H_6O_4$ (154.0266) (No. 35; VL + ActSyr + SyrAld + GUA NEG, Table S4)			
$C_7H_8O_4$ (156.0423) (No. 32; VL + ActSyr + SyrAld + GUA POS, Table S4)				46	$C_9H_{10}O_4$ (182.0579)	5	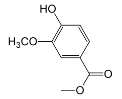
$C_{11}H_{12}O_7$ (256.0583) (No. 29; VL + ActSyr + SyrAld + GUA POS, Table S4)				$C_7H_6O_7$ (202.0113) (No. 16; VL + ActSyr + SyrAld NEG, Table S2)			
$C_8H_{10}O_5$ (186.0528) (No. 33; VL + ActSyr + SyrAld + GUA POS, Table S4)				$C_9H_8O_7$ (228.0270) (No. 17; VL + ActSyr + SyrAld NEG, Table S2)			
$C_{13}H_{14}O_8$ (298.0689) (No. 7; VL + ActSyr + SyrAld POS, Table S2)				$C_7H_8O_4$ (156.0423) (No. 32; VL + ActSyr + SyrAld + GUA POS, Table S4)			
$C_8H_6O_6$ (198.0164) (No. 25; VL* NEG, Table S3)				47	$C_9H_{10}O_5$ (198.0528)	5	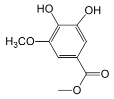
44	$C_{12}H_{10}O_7$ (266.0427)	8	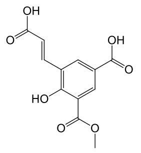	$C_8H_8O_7$ (216.0270) (No. 36; VL + ActSyr + SyrAld + GUA NEG, Table S4)			
$C_7H_6O_4$ (154.0266) (No. 35; VL + ActSyr + SyrAld + GUA NEG, Table S4)				$C_7H_6O_6$ (186.0164) (No. 41; DMB + ActSyr + SyrAld NEG, Table S5)			
45	$C_8H_{10}O_6$ (202.0477)	4	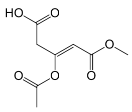	48	$C_{13}H_{12}O_4$ (232.0736)	8	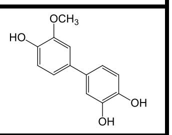

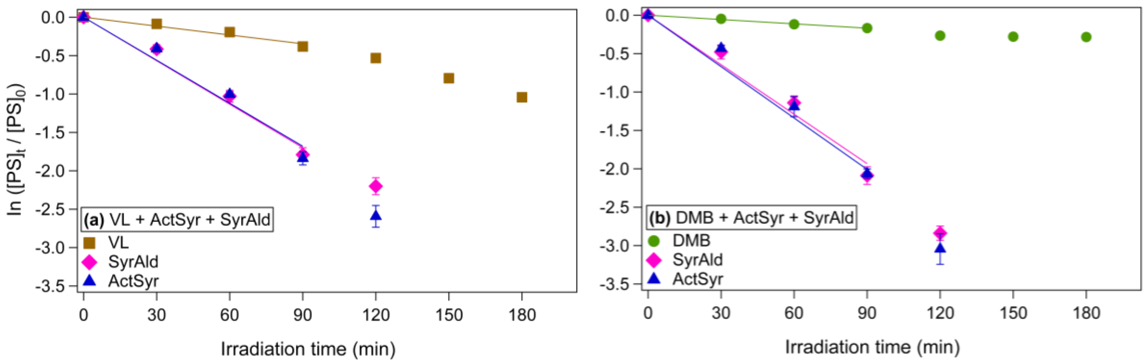

Figure S1. Representative kinetic plots from the irradiation of mixed photosensitizer (PS) systems. (a) The decay of VL, SyrAld, and ActSyr during the irradiation of VL + ActSyr + SyrAld. (b) The decay of DMB, SyrAld, and ActSyr during the irradiation of DMB + ActSyr + SyrAld. Note that ActSyr and SyrAld were fully consumed after 120 min of irradiation, similar to an earlier work.[23] Error bars represent 1 standard deviation; most error bars are smaller than the markers.

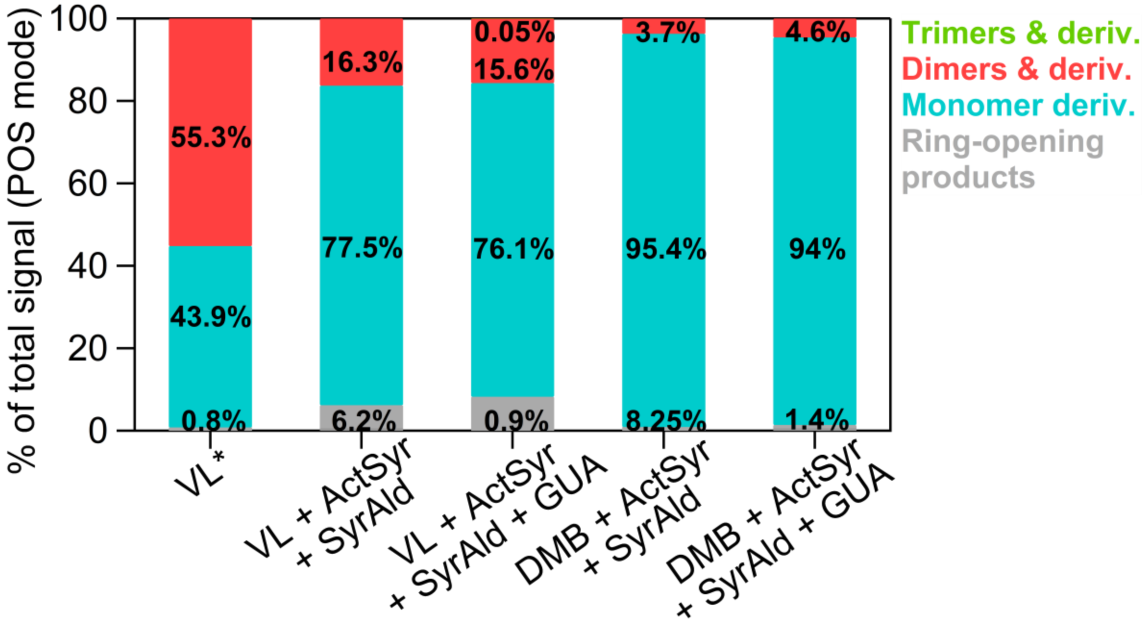

Figure S2. Signal-weighted distributions of VL*, VL + ActSyr + SyrAld, VL + ActSyr + SyrAld + GUA, DMB + ActSyr + SyrAld, and DMB + ActSyr + SyrAld + GUA aqSOA. These product distributions were calculated from UHPLC-HESI-Orbitrap-MS data obtained in the positive (POS) ion mode. The values indicate the contribution of different product classifications to the total signals for each reaction.

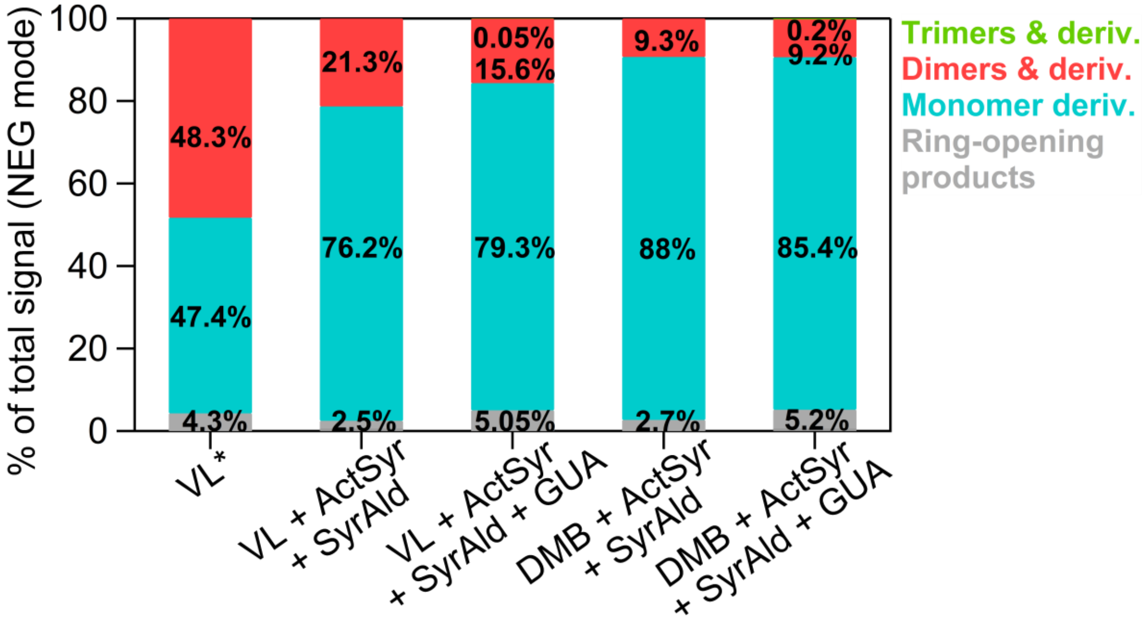

Figure S3. Signal-weighted distributions of VL*, VL + ActSyr + SyrAld, VL + ActSyr + SyrAld + GUA, DMB + ActSyr + SyrAld, and DMB + ActSyr + SyrAld + GUA aqSOA. These product distributions were calculated from UHPLC-HESI-Orbitrap-MS data obtained in the negative (NEG) ion mode. The values indicate the contribution of different product classifications to the total signals for each reaction.

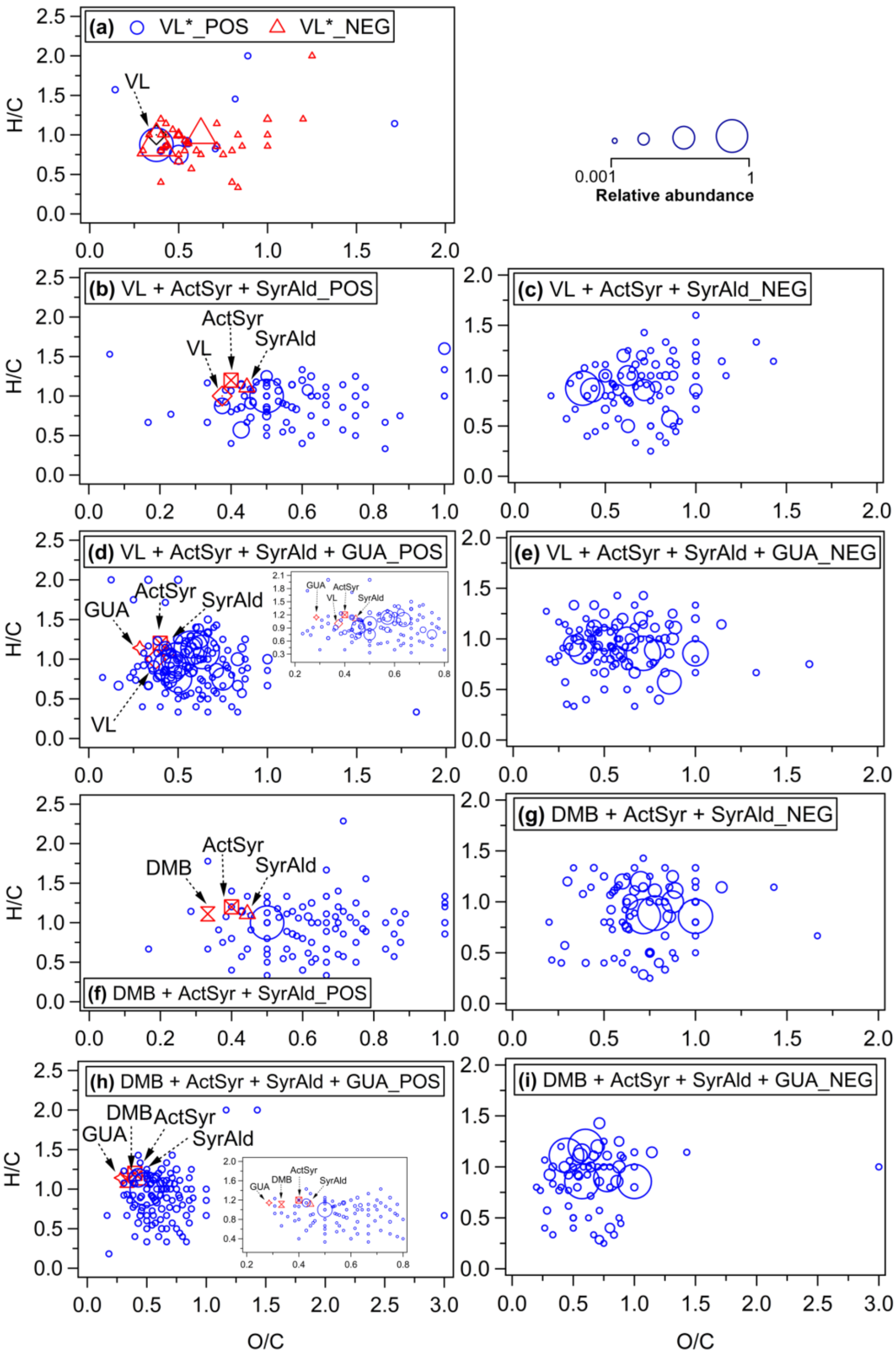

Figure S4. Van Krevelen diagrams of (a) VL*, (b, c) VL + ActSyr + SyrAld, (d, e) VL + ActSyr + SyrAld + GUA, (f, g) DMB + ActSyr + SyrAld, and (h, i) DMB + ActSyr + SyrAld + GUA aqSOA for positive (POS) and negative (NEG) ion modes. The marker size reflects the relative abundance in the sample. The markers for VL, ActSyr, SyrAld, DMB, and GUA are shown in POS mode panels (black marker in a and red markers in b, d, f, and h). The insets are expanded views of crowded sections. Note the different scales on the axes.

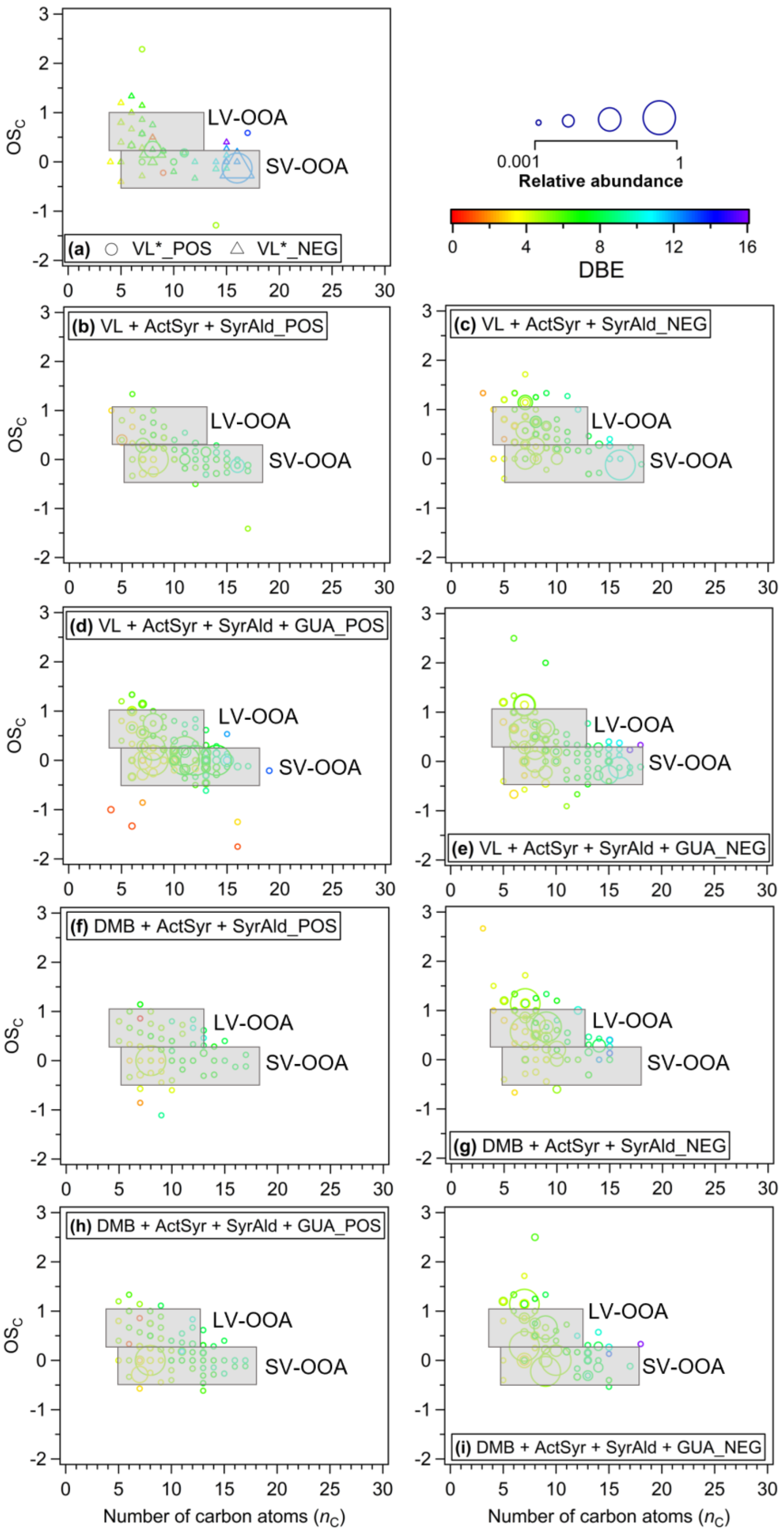

Figure S5. Plots of the OS_C vs. the number of carbon atoms (n_C) of (a) VL*, (b, c) VL + ActSyr + SyrAld, (d, e) VL + ActSyr + SyrAld + GUA, (f, g) DMB + ActSyr + SyrAld, and (h, i) DMB + ActSyr + SyrAld + GUA aqSOA for positive (POS) and negative (NEG) ion modes, colored by the DBE values. The marker size reflects the relative abundance in the sample. The shaded areas indicate the regions corresponding to low-volatility oxygenated organic aerosol (LV-OOA) and semivolatile oxygenated organic aerosol (SV-OOA).[13]

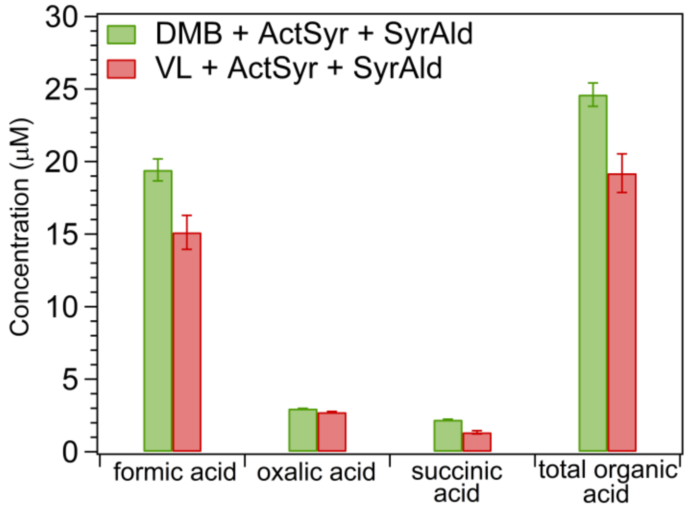

Figure S6. The concentration of formic, oxalic, succinic, and total organic acid for DMB + ActSyr + SyrAld and VL + ActSyr + SyrAld aqSOA. The total organic acid pertains to the sum of formic, oxalic, and succinic acid. Error bars represent one standard deviation of triplicate experiments.

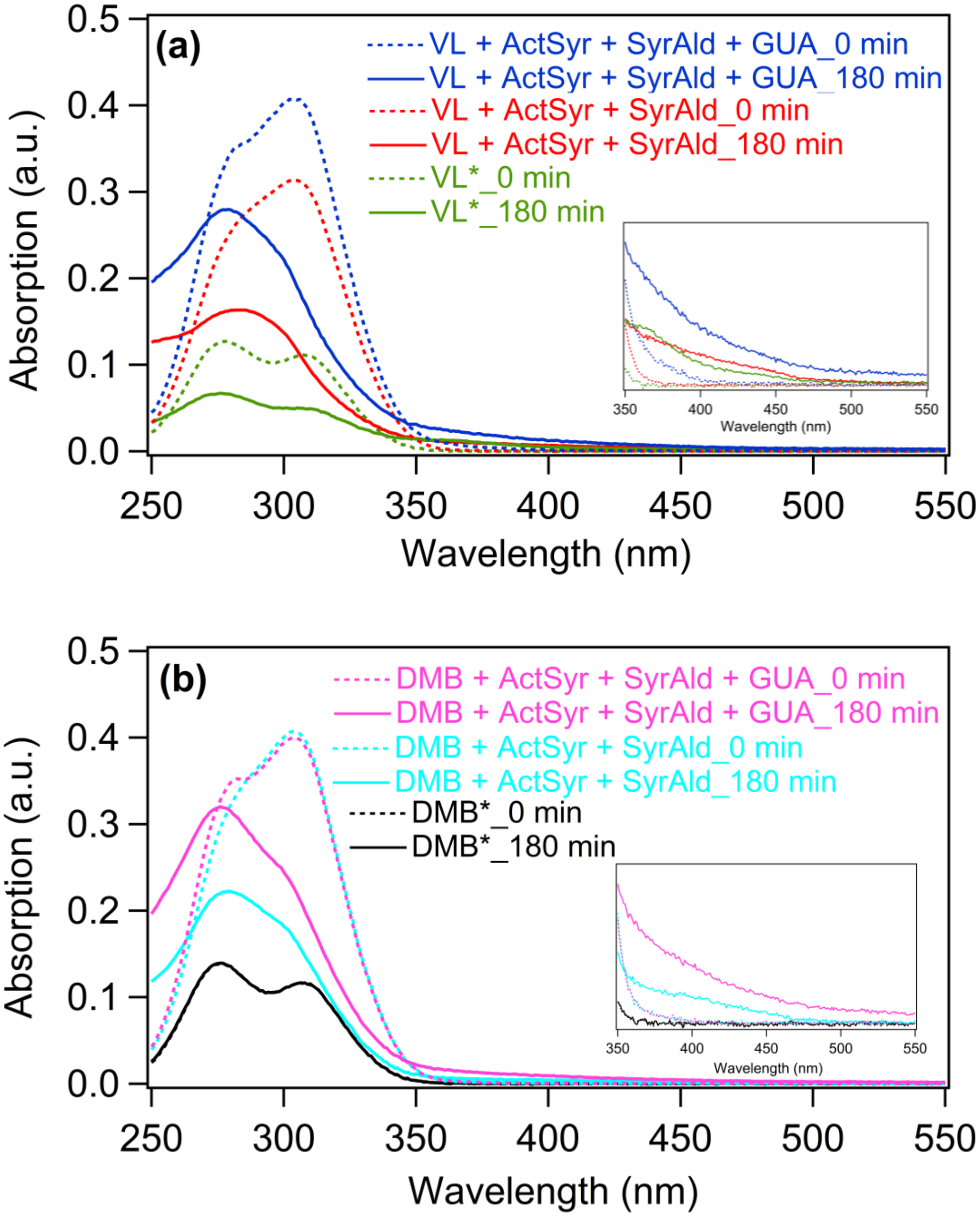

Figure S7. UV-Vis absorption spectra of (a) VL + ActSyr + SyrAld + GUA, VL + ActSyr + SyrAld, and VL* and (b) DMB + ActSyr + SyrAld + GUA, DMB + ActSyr + SyrAld, and DMB* at 0 min (before irradiation) and after 180 min of irradiation. The insets are the expanded view from 350 to 550 nm.

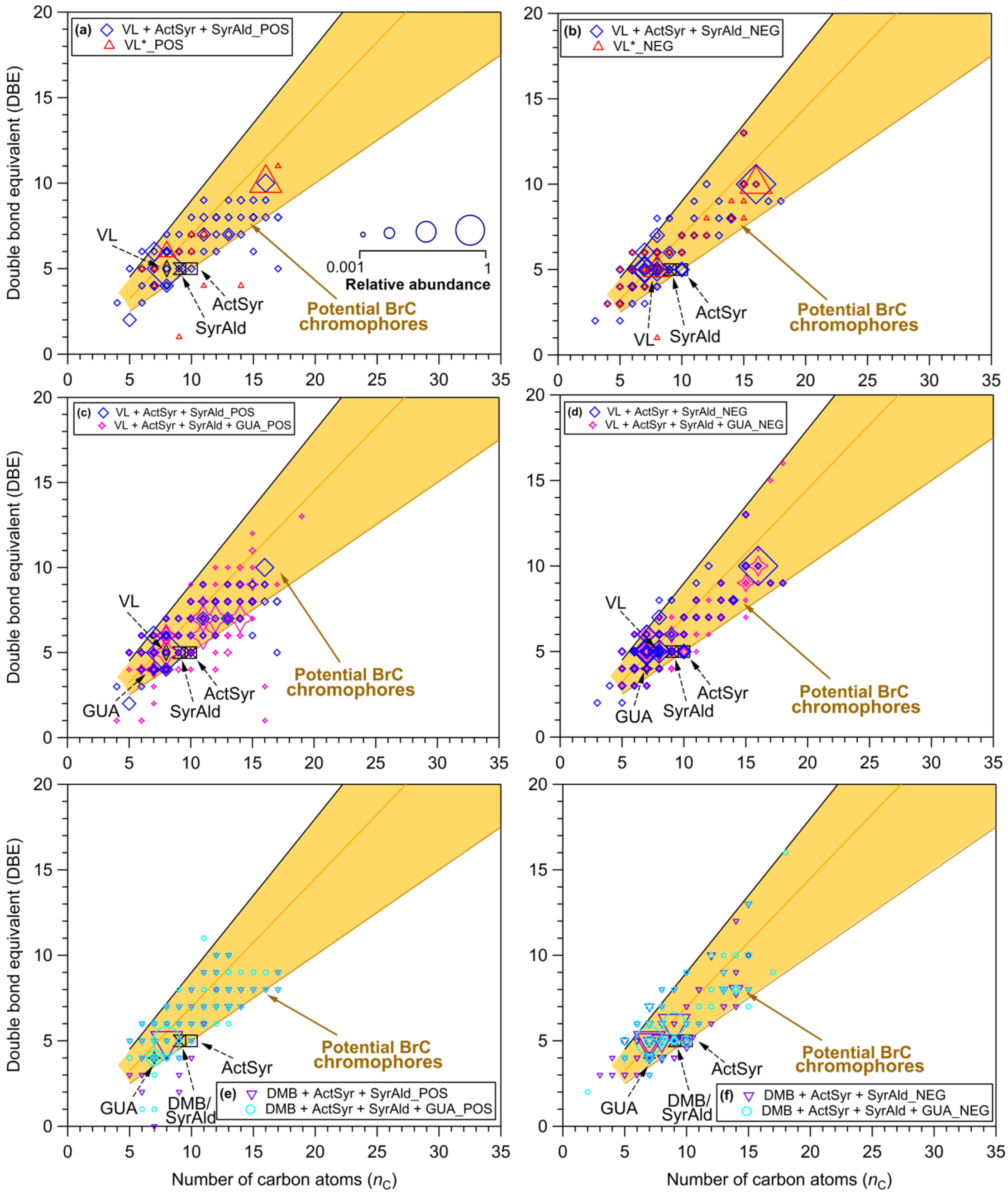

Figure S8. Plots of the DBE values vs. n_C[24] of (a, b) VL + ActSyr + SyrAld and VL*, (c, d) VL + ActSyr + SyrAld and VL + ActSyr + SyrAld + GUA, and (e, f) DMB + ActSyr + SyrAld and DMB + ActSyr + SyrAld + GUA aqSOA for positive (POS) and negative (NEG) ion modes. The marker size reflects relative abundance in the sample. The three lines indicate DBE reference values of fullerene-like hydrocarbons (top, black line),[25] cata-condensed polycyclic aromatic hydrocarbons (PAHs) (middle, orange line),[26] and linear conjugated polyenes (general formula C_xH_{x+2}; bottom, brown line). Species within the shaded area are potential BrC chromophores.

REFERENCES

(1) Laskin, J.; Laskin, A.; Nizkorodov, S. A.; Roach, P.; Eckert, P.; Gilles, M. K.; Wang, B.; Lee, H. J.; Hu, Q. Molecular selectivity of brown carbon chromophores. *Environ. Sci. Technol*. **2014**, *48*, 12047–12055.

(2) Yee, L. D.; Kautzman, K. E.; Loza, C. L.; Schilling, K. A.; Coggon, M. M.; Chhabra, P. S.; Chan, M. N.; Chan, A. W. H.; Hersey, S. P.; Crounse, J. D.; Wennberg, P. O.; Flagan, R. C.; Seinfeld, J. H. Secondary organic aerosol formation from biomass burning intermediates: phenol and methoxyphenols. *Atmos. Chem. Phys.* **2013**, *13*, 8019–8043.

(3) Li, Y. J.; Huang, D. D.; Cheung, H. Y.; Lee, A. K. Y.; Chan, C. K. Aqueous-phase photochemical oxidation and direct photolysis of vanillin - a model compound of methoxy phenols from biomass burning. *Atmos. Chem. Phys.* **2014**, *14*, 2871–2885.

(4) Yu, L.; Smith, J.; Laskin, A.; Anastasio, C.; Laskin, J.; Zhang, Q. Chemical characterization of SOA formed from aqueous-phase reactions of phenols with the triplet excited state of carbonyl and hydroxyl radical. *Atmos. Chem. Phys.* **2014**, *14*, 13801–13816.

(5) Yu, L.; Smith, J.; Laskin, A.; George, K. M.; Anastasio, C.; Laskin, J.; Dillner, A. M.; Zhang, Q. Molecular transformations of phenolic SOA during photochemical aging in the aqueous phase: competition among oligomerization, functionalization, and fragmentation. *Atmos. Chem. Phys.* **2016**, *16*, 4511–4527.

(6) He, L.; Schaefer, T.; Otto, T.; Kroflič, A.; Herrmann, H. Kinetic and theoretical study of the atmospheric aqueous-phase reactions of OH radicals with methoxyphenolic compounds. *J. Phys. Chem. A.* **2019**, *123*, 7828–7838.

(7) Chen, Y.; Li, N.; Li, X.; Tao, Y.; Luo, S.; Zhao, Z.; Ma, S.; Huang, H.; Chen, Y.; Ye, Z.; Ge, X. Secondary organic aerosol formation from $^3C^*$-initiated oxidation of 4-ethylguaiacol in atmospheric aqueous-phase. *Sci. Total Environ.* **2020,** *723*, 137953.

(8) Jiang, W.; Misovich, M. V.; Hettiyadura, A. P. S.; Laskin, A.; McFall, A. S.; Anastasio, C.; Zhang, Q. Photosensitized reactions of a phenolic carbonyl from wood combustion in the aqueous phase—chemical evolution and light absorption properties of aqSOA. *Environ. Sci. Technol.* **2021**, *55*, 5199–5211.

(9) Misovich, M. V.; Hettiyadura, A. P. S.; Jiang, W.; Zhang, Q.; Laskin, A. Molecular-level study of the photo-oxidation of aqueous-phase guaiacyl acetone in the presence of $^3C^*$: formation of brown carbon products. *ACS Earth Space Chem*, **2021**, *5*, 1983–1996.

(10) Mabato, B. R. G.; Lyu, Y.; Ji, Y.; Li, Y. J.; Huang, D. D.; Li, X.; Nah, T.; Lam, C. H.; Chan, C. K. Aqueous secondary organic aerosol formation from the direct photosensitized oxidation of vanillin in the absence and presence of ammonium nitrate. *Atmos. Chem. Phys.* **2022**, *22*, 273–293.

(11) Mabato, B. R. G.; Li, Y. J.; Huang, D. D.; Wang, Y.; Chan, C. K. Comparison of aqueous secondary organic aerosol (aqSOA) product distributions from guaiacol oxidation by non-phenolic and phenolic methoxybenzaldehydes as photosensitizers in the absence and presence of ammonium nitrate. *Atmos. Chem. Phys.* **2023**, *23*, 2859–2875.

(12) Koch, B. P.; Dittmar, T. From mass to structure: an aromaticity index for high-resolution mass data of natural organic matter. *Rapid Commun. Mass Spectrom,* **2006**, *20,* 926–932.

(13) Kroll, J. H.; Donahue, N. M.; Jimenez, J. L.; Kessler, S. H.; Canagaratna, M. R.; Wilson, K. R.; Altieri, K. E.; Mazzoleni, L. R.; Wozniak, A. S.; Bluhm, H.; Mysak, E. R.; Smith, J. D.; Kolb, C. E.; Worsnop, D. R. Carbon oxidation state as a metric for describing the chemistry of atmospheric organic aerosol. *Nat. Chem.* **2011**, *3*, 133–139.

(14) Kroll, J. H.; Lim, C. Y.; Kessler, S. H.; Wilson, K. R. Heterogeneous oxidation of atmospheric organic aerosol: kinetics of changes to the amount and oxidation state of particle-phase organic carbon. *J. Phys. Chem. A.* **2015**, *119*, 10767–10783.

(15) Lv, J.; Zhang, S.; Luo, L.; Cao, D. Solid-phase extraction-stepwise elution (SPE-SE) procedure for isolation of dissolved organic matter prior to ESI-FT-ICR-MS analysis. *Anal. Chim. Acta.* **2016**, *948*, 55–61.
(16) Bateman, A. P.; Laskin, J.; Laskin, A.; Nizkorodov, S. A. Applications of high-resolution electrospray ionization mass spectrometry to measurements of average oxygen to carbon ratios in secondary organic aerosols. *Environ. Sci. Technol.* **2012**, *46*, 8315–832.
(17) Nguyen, T. B.; Lee, P. B.; Updyke, K. M.; Bones, D. L.; Laskin, J.; Laskin, A.; Nizkorodov, S. A. Formation of nitrogen- and sulfur-containing light-absorbing compounds accelerated by evaporation of water from secondary organic aerosols. *J. Geophys. Res.: Atmos.* **2012**, *117*, D01207.
(18) Powelson, M. H.; Espelien, B. M.; Hawkins, L. N.; Galloway, M. M.; De Haan, D. O. Brown carbon formation by aqueous-phase carbonyl compound reactions with amines and ammonium sulfate. *Environ. Sci. Technol*, **2014**, *48*, 985–993.
(19) Lignell, H.; Epstein, S. A.; Marvin, M. R.; Shemesh, D.; Gerber, B.; Nizkorodov, S. Experimental and theoretical study of aqueous cis-pinonic acid photolysis. *J. Phys. Chem. A.* **2013**, *117*, 12930–12945.
(20) Smith, J. D.; Kinney, H.; Anastasio, C. Phenolic carbonyls undergo rapid aqueous photodegradation to form low-volatility, light-absorbing products. *Atmos. Environ.* **2016**, *126*, 36–44.
(21) Wang, Y.; Qiu, T.; Zhang, C.; Hao, T.; Mabato, B. R. G.; Zhang, R.; Gen, M.; Chan, M. N.; Huang, D. D.; Ge, X.; Wang, J.; Du, L.; Huang, R.; Chen, Q.; Hoi, K. I.; Mok, K. M.; Chan, C. K.; Li, Y. Co-photolysis of mixed chromophores affects atmospheric lifetimes of brown carbon. *Environ. Sci.: Atmos.* **2023**, *3*, 1145–1158.
(22) Finlayson-Pitts, B. J., Pitts, J. N. Chapter 3 - Spectroscopy and Photochemistry: Fundamentals. In *Chemistry of the Upper and Lower Atmosphere*; Finlayson-Pitts, B. J., Pitts, J. N., Eds.; Academic Press: San Diego 2000; pp 43–85.
(23) Huang, D. D.; Zhang, Q.; Cheung, H. H. Y.; Yu, L.; Zhou, S.; Anastasio, C.; Smith, J. D.; Chan, C. K. Formation and evolution of aqSOA from aqueous-phase reactions of phenolic carbonyls: comparison between ammonium sulfate and ammonium nitrate solutions. *Environ. Sci. Technol.* **2018**, *52*, 9215–9224.
(24) Lin, P.; Fleming, L. T.; Nizkorodov, S. A.; Laskin, J.; Laskin, A. Comprehensive molecular characterization of atmospheric brown carbon by high resolution mass spectrometry with electrospray and atmospheric pressure photoionization. *Anal. Chem.* **2018**, *90,* 12493–12502.
(25) Lobodin, V. V.; Marshall, A. G.; Hsu, C. S. Compositional space boundaries for organic compounds. *Anal. Chem.* **2012**, *84*, 3410–3416.
(26) Siegmann, K.; Sattler, K. Formation mechanism for polycyclic aromatic hydrocarbons in methane flames. *J. Chem. Phys.* **2000**, *112*, 698–709.
